# Prediction of Percutaneous Coronary Intervention Success in Patients With Moderate to Severe Coronary Artery Calcification Using Machine Learning Based on Coronary Angiography: Prospective Cohort Study

**DOI:** 10.2196/70943

**Published:** 2025-07-11

**Authors:** Zixiang Ye, Zhangyu Lin, Enmin Xie, Chenxi Song, Rui Zhang, Hao-Yu Wang, Shanshan Shi, Lei Feng, Kefei Dou

**Affiliations:** 1 Department of Cardiology, Fuwai Hospital, Chinese Academy of Medical Sciences & Peking Union Medical College, No 167 North Lishi Road, Xicheng District, Beijing, 100037, China, 86 88398866; 2 State Key Laboratory of Cardiovascular Disease, Beijing, China; 3 Cardiometabolic Medicine Center, Fuwai Hospital, Chinese Academy of Medical Sciences & Peking Union Medical College, Beijing, China

**Keywords:** Extreme Gradient Boosting, artificial intelligence, coronary angiography, percutaneous coronary intervention, coronary artery calcification

## Abstract

**Background:**

Given the challenges faced during percutaneous coronary intervention (PCI) for heavily calcified lesions, accurately predicting PCI success is crucial for enhancing patient outcomes and optimizing procedural strategies.

**Objective:**

This study aimed to use machine learning (ML) to identify coronary angiographic vascular characteristics and PCI procedures associated with the immediate procedural success rates of PCI in patients exhibiting moderate to severe coronary artery calcification (MSCAC).

**Methods:**

This study included patients who underwent PCI between January 2017 and December 2018 in a cardiovascular hospital, comprising 3271 patients with MSCAC and 17,998 with no or mild coronary artery calcification. Six ML models—k-nearest neighbor, gradient boosting decision tree, Extreme Gradient Boosting (XGBoost), logistic regression, random forest, and support vector machine—were developed and validated, with synthetic minority oversampling technique used to address imbalance data. Model performance was compared using multiple parameters, and the optimal algorithm was selected. Model interpretability was facilitated by Shapley Additive Explanations (SHAP), identifying the top 6 coronary angiographic features with the highest SHAP values. The importance of different PCI procedures was also elucidated via SHAP values. Testing validation was performed in a separate cohort of 1437 patients with MSCAC in 2013. External validation was conducted in a general hospital of 204 patients with MSCAC in 2021. Sensitivity analyses were conducted in patients with acute coronary syndrome and chronic coronary syndrome.

**Results:**

In the development cohort, 7.6% (n=248) of patients with MSCAC experienced PCI failure compared to 4.3% (n=774) of patients with no or mild coronary artery calcification. The XGBoost model demonstrated superior performance, achieving the highest area under the receiver operator characteristic curve (AUC) of 0.984, average precision (AP) of 0.986, *F*_1_-score of 0.970, and G-mean of 0.970. Calibration curves indicated reliable predictive accuracy. The key predictive factors identified included lesion length, minimum lumen diameter, thrombolysis in myocardial infarction flow grade, chronic total occlusion, reference vessel diameter, and diffuse lesion (SHAP value 1.65, 1.40, 0.92, 0.60, 0.54, and 0.47, respectively). The use of modified balloons for calcified lesions had a positive effect on PCI success in patients with MSCAC (SHAP value 0.16). Sensitivity analyses showed consistent model performance across subgroups with similar top 5 coronary angiographic variables. The optimized XGBoost model maintained robust predictive performance in the testing cohort, with an AUC of 0.972, AP of 0.962, and *F*_1_-score of 0.940, and in the external validation set, with an AUC of 0.810, AP of 0.957, and *F*_1_-score of 0.892.

**Conclusions:**

This study successfully revealed the important PCI failure risk factors, such as lesion length and modified balloons, using ML models to help clinicians manage PCI strategies in patients with complex coronary artery disease such as MSCAC.

## Introduction

Coronary artery calcification (CAC) is associated with poor prognosis in patients with coronary artery disease (CAD) [1,2]. Patients with moderate to severe coronary artery calcification (MSCAC) have a significantly higher incidence of major adverse cardiovascular events (MACEs) compared to those with milder calcification [3,4]. During percutaneous coronary intervention (PCI) for heavily calcified coronary lesions, the manipulation of guidewires and the deployment of balloons or stents are notably more challenging than in normal coronary arteries. Balloon inflation may be insufficient in the calcified lesions, and there is a risk of balloon rupture. Typically, higher pressures are required to expand calcified lesions, which significantly increases the likelihood of complications such as vascular dissection, perforation, rupture, and no-reflow leading to a higher failure rate of PCI in patients with MSCAC [5].

Coronary angiography (CAG) is a routine examination before PCI, providing clear visualization of the morphological characteristics of coronary vessels and allowing for the assessment of the degree of coronary calcification [6]. It is considered the gold standard for diagnosing coronary stenosis [7]. Therefore, the ability to predict the difficulty and success rate of PCI procedures through coronary angiographic imaging is crucial for both patients with MSCAC and cardiologists. Currently, there is less research available that predicts the difficulty and success rate of PCI operations based on the characteristics of vessels observed in CAG for patients with MSCAC.

Machine learning (ML) technology is a cutting-edge advancement in the rapidly evolving field of artificial intelligence, with numerous applications in medicine [8]. Compared to traditional statistical methods, ML can provide better clinical prediction accuracy and performance, as well as quicker processing rates, and has a better ability to accurately identify the most effective predictors of clinical outcomes [9]. ML has been widely applied in the cardiovascular field, including the prediction of fractional flow reserve after PCI, estimation of computed tomography–derived fractional flow reserve, automated quantification of myocardial blood flow and ischemic myocardial volume percentage, as well as the prediction of adverse events following acute coronary syndrome (ACS) [10-13]. However, its application in exploring the relationship between CAG imaging features or PCI procedures and the success rate of PCI in patients with MSCAC has been limited.

The purpose of this research is to use ML techniques to explore the coronary angiographic vascular characteristics and PCI procedures most closely associated with the immediate procedural success rate of PCI in patients with MSCAC, aiming to achieve precise predictions of immediate success rates for PCI in this population.

## Methods

### Ethical Considerations

This study was approved by the ethics committee of Fuwai Hospital, Chinese Academy of Medical Sciences (approval 2016‐847 and 2013‐449) and complied with the Helsinki Principles. The enrolled patients gave informed consent for their treatment. Participants were assured that their privacy and confidentiality would be strictly protected, and all data were anonymized prior to analysis. No personal identifying information was collected or stored.

### Study Design

This study was a retrospective analysis of 2 prospective clinical studies at Fuwai Hospital. The development cohort consecutively included patients who underwent PCI from January 2017 to December 2018, and the testing cohort included patients who underwent PCI from January 2013 to December 2013 (Tables S1 and S2 in Multimedia Appendix 1). The predictive performance of the ML model was also validated in an external validation cohort, which consisted of patients with CAD and MSCAC who underwent PCI at China-Japan Friendship Hospital in 2021 (Table S3 in Multimedia Appendix 1).

**Figure 1.**
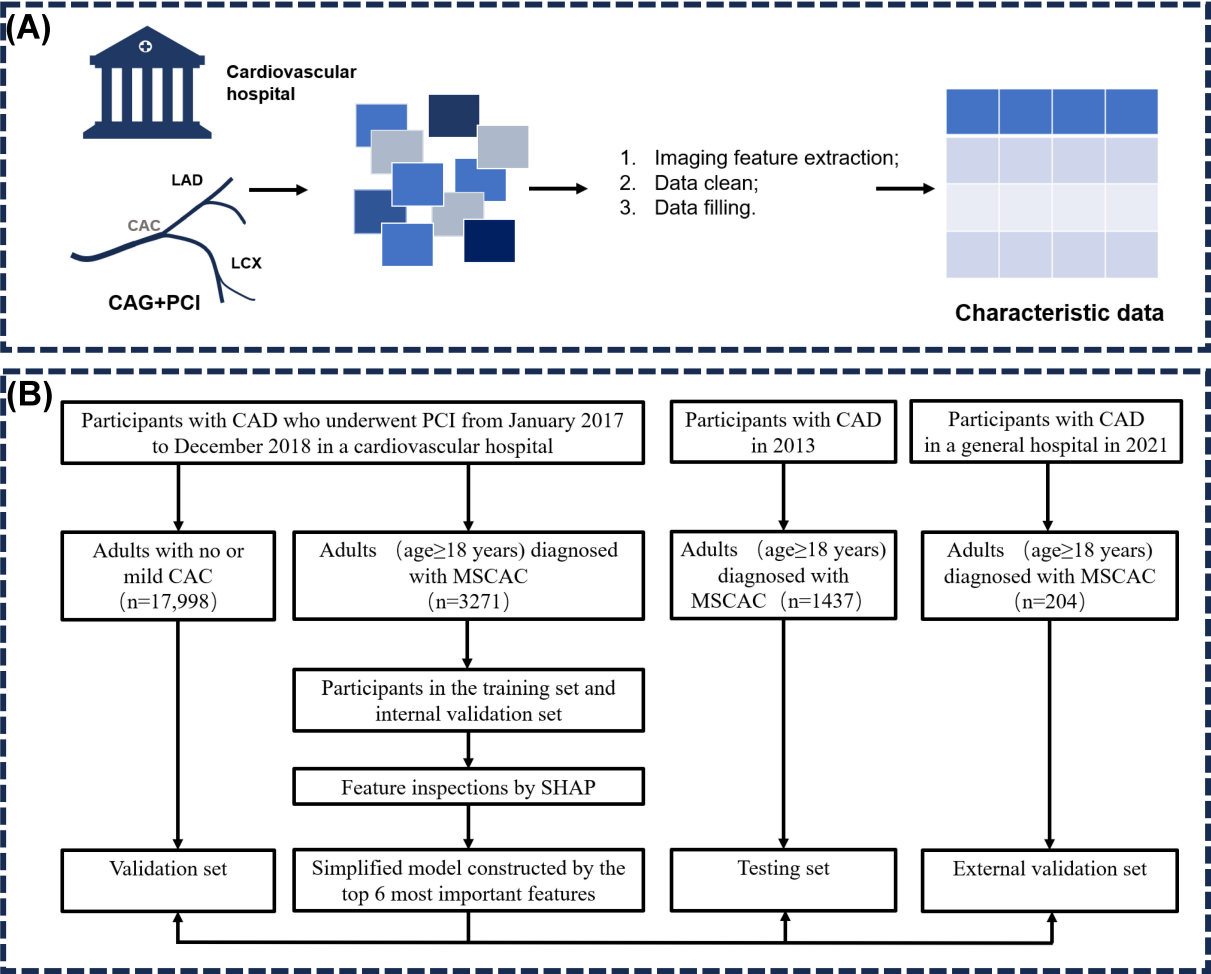
Overview of study design and workflow. (A) Dataset construction of patients with CAD. (B) Flowchart of the study design and patient selection. CAC: coronary artery calcification; CAD: coronary artery disease; CAG: coronary angiography; LAD: left anterior descending branch; LCX: left circumflex branch; MSCAC: moderate to severe coronary artery calcification; PCI: percutaneous coronary intervention; SHAP: Shapley Additive Explanations.

In this study, the top 6 CAG imaging features, which were most associated with the success of immediate PCI treatment, were selected based on the variable importance ranking indicated by the Shapley Additive Explanations (SHAP) values derived from the ML algorithm from the development cohort and validated in patients with mild CAC or without CAC in the testing cohort and the external validation cohort (Figure 1). Additionally, we further explored the relationship between different PCI procedures and PCI success rates in patients with MSCAC compared to those with no or mild CAC. Detailed descriptions of both studies are available in previous papers [14,15].

The inclusion criteria were (1) a patient with CAD with calcification interpretation data based on CAG by 2 experienced cardiologists and (2) MSCAC was identified. The exclusion criteria were (1) stenting before coronary intervention affected the interpretation of the degree of calcification, (2) previous coronary angioplasty bypass grafting treatment, and (3) missing data greater than 30%. The detailed inclusion and exclusion criteria for the cohort are presented in Figures S1-S3 in Multimedia Appendix 2.

The choice of puncture position, interventional strategy, and medical devices for all PCIs was determined by the operator. The use of perioperative antiplatelet and anticoagulant medications follows the operator’s judgment and the recommendations of the latest guidelines [16].

### PCI Procedure Success and Coronary Calcification

The definition of immediate PCI procedure success was a restoration of grade 2 or 3 thrombolysis in myocardial infarction (TIMI) flow with residual stenosis (RS) of less than 50% as well as without significant operational complications, such as intraoperative stent thrombosis, coronary artery dissection, perforation of the coronary artery, and in-hospital MACE [17].

Coronary calcification is defined as a heterogeneous high-density image along the course of the vessel observed under fluoroscopy without the administration of contrast [18]. Based on the findings from CAG, the severity of calcified lesions can be categorized as follows: (1) no calcification, which means no significant coronary calcification is detected during angiography; (2) mild calcification, defined as a faint and indistinct high-density shadow only visible during cardiac motion, with no evidence of calcification when the heart is at rest; (3) moderate coronary calcification, characterized by a clear, dense calcified shadow visible during cardiac beats; and (4) severe coronary calcification, defined as a clear, dense calcified shadow visible with or without cardiac motion [19]. Each CAG image was judged and confirmed by more than 2 cardiologists [20].

### Predictor Variables

Demographic data and CAG as well as interventional information of each patient were collected for this study. Demographic characteristics included sex, age, BMI, smoking, and comorbidities. Coronary angiographic imaging features (including dominant branch, bifurcation lesion, angulation, calcification degree, lesion length, minimum diameter of lesion lumen, lesion diffuseness, chronic total occlusion [CTO], and extreme tortuosity) were interpreted and recorded by experienced cardiovascular physicians, as described in previous studies [14,15]. Parameters such as lesion length, minimum diameter of the lesion lumen, and reference vessel diameter (RVD) were measured by experienced operators using the angiographic catheter as a reference, based on CAG images. Additionally, details of coronary PCI treatment were recorded by skilled cardiologists involved in the coronary intervention. Figure 1A illustrates the CAG characteristic data and the PCI treatment data extraction procedure.

### Imbalance Data Processing

PCI intervention failures are a relatively small percentage compared to the total number of PCI procedures, so our data are imbalanced. Imbalanced data mean that only a small percentage of instances are used. Thus, before training each of our models, we used the synthetic minority over-sampling technique (SMOTE) [21] to rebalance the classes. SMOTE uses a variant of the k-nearest neighbor (KNN) technique to synthesize new minority class instances from pre-existing minority instances [22]. To avoid performance evaluation bias, the SMOTE algorithm was applied only to the training set of the data. To establish a balanced training set, the SMOTE algorithm subsequently engages in oversampling the minority instances, comprising both original and synthesized samples, while simultaneously undertaking undersampling of the majority class. Furthermore, to prevent data leaking through the use of the R *mice* package, missing values were imputed independently using a fully conditional specification [23].

### ML Training and Tuning

We developed 6 classification algorithms based on the CAG characteristics: KNN, gradient boosting decision tree machine (GBDT), Extreme Gradient Boosting (XGBoost), logistic regression (LR), random forest, and support vector machine. These classifiers were chosen for their potential strong performance with imbalanced data categorization, scalability, and resilience. In total, 30% of the patients in the development cohort were removed for the internal validation set and 70% of the patients for the algorithm development set.

The hyperparameter optimization process for XGBoost involves systematically tuning key parameters [24]. Ten-fold cross-validation was used to train and fine-tune each of the aforementioned models (10 repetitions). The training data are divided into 10 random samples for this validation approach. The model is then trained on the remaining groups after each sample is held out in turn. Confusion matrices are built using the model’s predictions in the held-out group, and an optimization measure is determined. During the validation procedure, the optimal model is chosen using the optimization metric to avoid potential overfitting. We also used an adaptive resample technique to adjust our models’ hyperparameters [25].

### Statistical Analysis

Categorical variables are presented as counts with percentages, and continuous variables are reported as medians with IQRs. The chi-square test, the Fisher exact test, or the Wilcoxon rank sum test were used to compare different attributes. A 2-sided *P* value below .05 was considered statistically significant. All statistical analyses and calculations were conducted using R software (version 4.1.3; R Foundation for Statistical Computing) and Python (version 3.9.12; Python Software Foundation).

To mitigate the impact of imbalanced data on model recommendations, this study used multiple performance metrics to evaluate and compare different ML models, including the area under the receiver operator characteristic curve (AUC), average precision (AP), sensitivity, specificity, *F*_1_-score, negative predictive value, positive predictive value, and G-mean. The classifier demonstrating optimal performance was selected for the final model. Calibration curves and prediction probability histograms were used for the re-evaluation of the best-performing model. Furthermore, to evaluate the utility of the models for decision-making, we conducted decision curve analysis (DCA) to quantify the net benefit at various threshold probabilities. Additionally, 5-knotted multivariate restricted cubic spline regression was performed to assess the nonlinear or linear association between the most important predictors and PCI immediate success.

The interpretation of the predictive model is facilitated by SHAP, a comprehensive methodology designed to accurately quantify the contribution and influence of each feature on the final predictions, which has been used widely in the cardiovascular realm [26]. SHAP values elucidate the extent to which each predictor contributes, whether positively or negatively, to the target variable. Furthermore, each observation within the dataset can be interpreted through its corresponding set of SHAP values, providing a detailed understanding of individual contributions to the model’s predictions [27].

## Results

### Baseline Characteristics

A total of 3271 patients with MSCAC underwent PCI in the 2017 to 2018 cohort according to the established inclusion and exclusion criteria (Table 1). Among them, 3023 (92.4%) patients had a successful PCI, while 248 (7.6%) patients experienced PCI failure. Patients who experienced PCI failure were generally older, had a more severe degree of calcification, higher Syntax scores, and greater vessel tortuosity, as well as more instances of total occlusion and CTO. These patients also had higher rates of TIMI 0 flow, more diffuse lesions, along with a smaller minimum lumen diameter (MLD) and RVD. In terms of PCI management, patients with MSCAC who experienced PCI failure typically had lower rates of the use of adjunctive strategies such as modified balloons, rotational atherectomy, postdilation, kissing balloon techniques, and balloon predilation, as well as a lower proportion of stent placement. Table S4 in Multimedia Appendix 1 shows PCI failure and reasons in both the development set and testing set.

**Table 1.** Baseline characteristics of patients with moderate to severe coronary artery calcification who underwent percutaneous coronary intervention (PCI) from 2017 to 2018.

	Overall (n=3271)	PCI success (n=3023)	PCI failure (n=248)	*P* value
Sex (male), n (%)	2421 (74)	2230 (73.8)	191 (77)	.30
Age (years), median (IQR)	62.20 (54.40-68.50)	62.10 (54.40-68.30)	63.10 (55.00-70.80)	.17
BMI, median (IQR)	25.46 (23.53-27.68)	25.40 (23.51-27.68)	25.74 (23.86-28.05)	.10
ACS^a^, n (%)	2060 (63)	1907 (63.1)	153 (61.7)	.71
T2DM^b^, n (%)	1119 (34.2)	1029 (34)	90 (36.3)	.52
Dyslipidemia, n (%)	2411 (73.7)	2221 (73.5)	190 (76.6)	.32
Family history CAD^c^, n (%)	356 (10.9)	329 (10.9)	27 (10.9)	>.99
Smoking, n (%)	2085 (63.7)	1919 (63.5)	166 (66.9)	.31
Heavily calcified, n (%)	657 (20.1)	570 (18.9)	87 (35.1)	<.001
LM^d^, n (%)	143 (4.4)	140 (4.6)	3 (1.2)	.02
LAD^e^, n (%)	1567 (47.9)	1469 (48.6)	98 (39.5)	.007
LCX^f^, n (%)	439 (13.4)	414 (13.7)	25 (10.1)	.13
RCA^g^, n (%)	1140 (34.9)	1017 (33.6)	123 (49.6)	<.001
Pre-syntax, median (IQR)	13.00 (8.00-21.00)	13.00 (7.00-20.00)	20.00 (13.00-27.50)	<.001
Triple vessel, n (%)	1781 (54.4)	1609 (53.2)	172 (69.4)	<.001
LM disease, n (%)	369 (11.3)	342 (11.3)	27 (10.9)	.92
MLD^h^ (mm), median (IQR)	0.29 (0.03-0.50)	0.30 (0.12-0.50)	0.00 (0.00-0.00)	<.001
RVD^i^ (mm), median (IQR)	3.00 (2.80-3.50)	3.00 (2.80-3.50)	3.00 (2.50-3.08)	.001
DS^j^ (%), median (IQR)	90.00 (80.00-99.00)	90.00 (80.00-95.00)	100.00 (100.00-100.00)	<.001
Pre-TIMI^k^, n (%)				<.001
0	729 (22.3)	538 (17.8)	191 (77)	
1	177 (5.4)	164 (5.4)	13 (5.2)	
2	394 (12)	385 (12.7)	9 (3.6)	
3	1971 (60.3)	1936 (64)	35 (14.1)	
B2_C_lesion, n (%)	2716 (83)	2480 (82)	236 (95.2)	<.001
Diffuse range, n (%)	2277 (69.6)	2076 (68.7)	201 (81)	<.001
Lesion length (mm), median (IQR)	30.00 (19.00-47.00)	30.00 (18.00-47.00)	30.00 (25.75-48.00)	.001
Concentric, n (%)	347 (10.6)	309 (10.2)	38 (15.3)	.02
Extremely tortuosity, n (%)	166 (5.1)	133 (4.4)	33 (13.3)	<.001
Angulated, n (%)	1199 (36.7)	1093 (36.2)	106 (42.7)	.045
Irregular, n (%)	2901 (88.7)	2664 (88.1)	237 (95.6)	.001
CTO^l^, n (%)	450 (13.8)	299 (9.9)	151 (60.9)	<.001
Ostial, n (%)	432 (13.2)	385 (12.7)	47 (19)	.007
Bifurcation, n (%)	596 (18.2)	566 (18.7)	30 (12.1)	.001
Prethrombosis, n (%)	156 (4.8)	128 (4.2)	28 (11.3)	<.001
Modified balloon, n (%)	340 (10.4)	332 (11)	8 (3.2)	<.001
Rotational atherectomy, n (%)	77 (2.4)	76 (2.5)	1 (0.4)	.06
Stent number, median (IQR)	2.00 (1.00-2.00)	2.00 (1.00-2.00)	2.00 (1.00-2.00)	.001
Postdilatation, n (%)	2518 (77)	2360 (78.1)	158 (63.7)	<.001
Tandem stent, n (%)	1502 (45.9)	1379 (45.6)	123 (49.6)	.25
IVUS^m^, n (%)	375 (11.5)	351 (11.6)	24 (9.7)	.42

a ACS: acute coronary syndrome.

b T2DM: type 2 diabetes mellitus.

c CAD: coronary artery disease.

d LM: left main coronary artery.

e LAD: left anterior descending branch.

f LCX: left circumflex branch.

g RCA: right coronary artery.

h MLD: minimal lumen diameter.

i RVD: reference vessel diameter.

j DS: degree of stenosis.

k TIMI: thrombolysis in myocardial infarction.

l CTO: chronic total occlusion.

m IVUS: intravascular ultrasound.

### CAG ML Model Establishment

**Figure 2.**
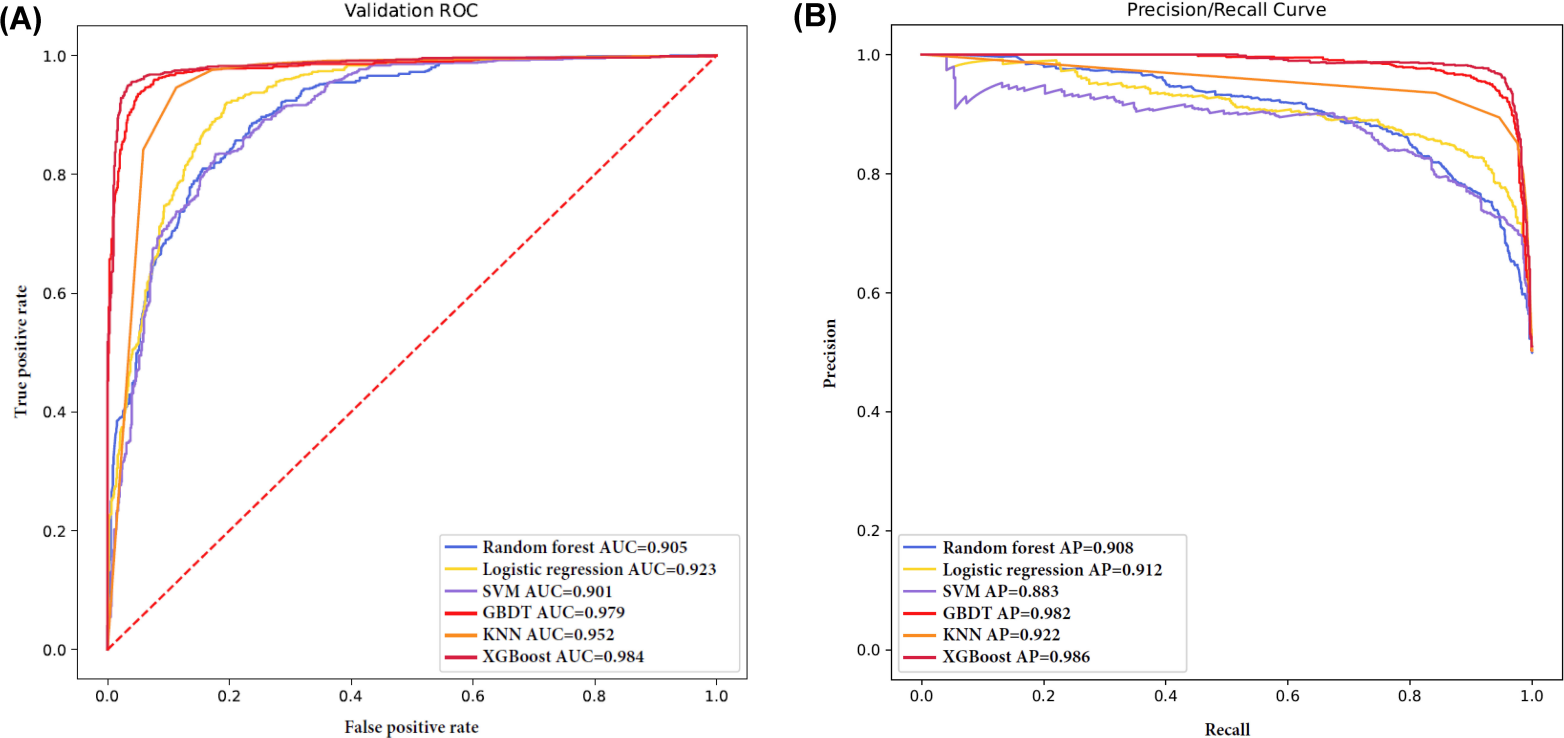
Various performances of ML models. (A) ROC of ML. (B) Precision/recall curve of ML. The XGBoost showed the best performance. AP: average precision; AUC: area under the receiver operator characteristic curve; GBDT: gradient boosting decision tree machine; KNN: k-nearest neighbor; ML: machine learning; ROC: receiver operating characteristic; SVM: support vector machine; XGBoost: Extreme Gradient Boosting.

The included patients with MSCAC were randomly divided in a 7:3 ratio into a model training cohort and an internal validation cohort. Six ML models were developed to predict immediate successful PCI in the model development cohort based on the CAG characteristic data, and their predictive performance was compared in the internal validation cohort. Among the models evaluated in the internal validation cohort, the XGBoost model demonstrated the highest predictive accuracy for immediate PCI success, achieving an AUC of 0.984 and an AP of 0.986. Figure 2 illustrates the discriminatory performance of these ML models, represented through receiver operating characteristic and precision-recall curves, following 10-fold cross-validation in the internal validation set. The calibration curve and predicted probability histogram indicate that the XGBoost model exhibits a certain level of reliability. The bimodal predicted probability histogram suggests that the model assigns probabilities close to either 0 or 1 for most samples, demonstrating relatively high-confidence decision-making and strong discriminative ability (Figure S4 in Multimedia Appendix 2). The GBDT model (AUC 0.979), KNN model (AUC 0.952), and XGBoost (AUC 0.984) demonstrated superior predictive capabilities for immediate PCI success in this patient population when compared to the traditional LR. Table 2 presents a comprehensive array of performance metrics for the various ML models evaluated in this study. Specific details (learning rate, tree depth, and so on) were described in Table S5 in Multimedia Appendix 1.

**Table 2.** Comparison of various machine learning models’ performance.

Machine learning	AUC^a^	Sensitivity	Specificity	PPV^b^	NPV^c^	AP^d^	*F*_1_-score	G-mean
RF^e^	0.905	0.836	0.804	0.812	0.831	0.908	0.834	0.821
LR^f^	0.923	0.896	0.818	0.832	0.886	0.912	0.863	0.856
SVM^g^	0.901	0.902	0.858	0.866	0.897	0.883	0.883	0.880
GBDT^h^	0.979	0.955	0.925	0.928	0.953	0.982	0.940	0.939
KNN^i^	0.952	0.976	0.828	0.852	0.971	0.922	0.909	0.899
XGBoost^j^	0.984	0.965	0.975	0.975	0.970	0.986	0.970	0.970

a AUC: area under the receiver operator characteristic curve.

b PPV: positive predictive value.

c NPV: negative predictive value.

d AP: average precision.

e RF: random forest.

f LR: logistic regression.

g SVM: support vector machine.

h GBDT: gradient boosting decision tree machine.

i KNN: k-nearest neighbor.

j XGBoost: Extreme Gradient Boosting.

### CAG XGBoost Model Explained by SHAP

**Figure 3.**
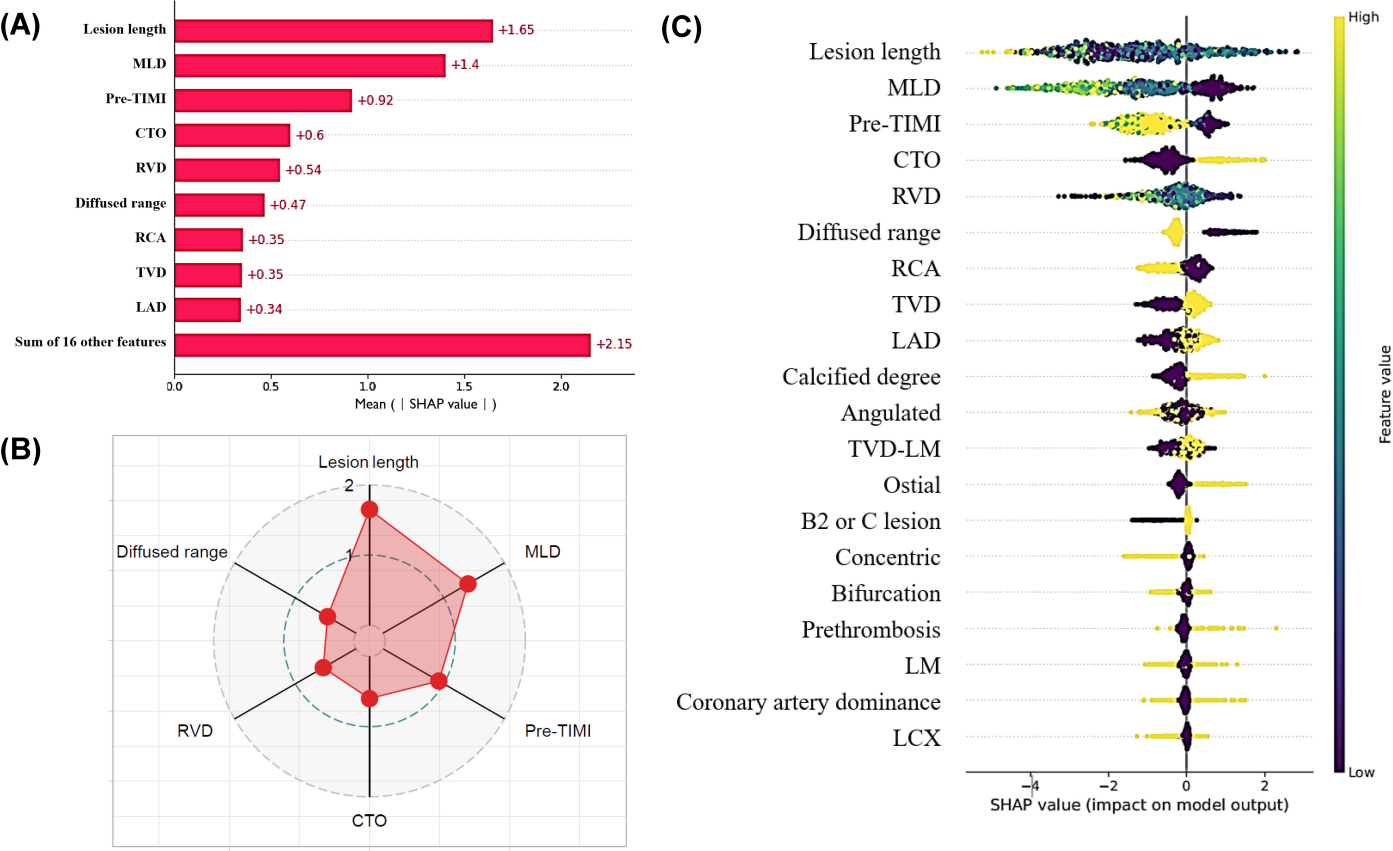
Visualizing the importance of various predictors by SHAP in patients with moderate to severe coronary artery calcification (MSCAC). (A and B) Bar chart and radar plot that rank the importance of the top 6 significant variables most associated with the PCI success rate in patients with MSCAC. (C) Impact of the top 20 features in the XGBoost model. CTO: chronic total occlusion; LAD: left anterior descending branch; LCX: left circumflex branch; LM: left main coronary artery; MLD: minimal lumen diameter; RCA: right coronary artery; RVD: reference vessel diameter; SHAP: Shapley Additive Explanations; TIMI: thrombolysis in myocardial infarction; TVD: triple vessel disease.

The SHAP algorithm was used to visually illustrate the significance of each factor contributing to the prediction of PCI success by the XGBoost model. Figure 3 displays the feature importance plot in descending order, highlighting the top 6 predictive factors for immediate PCI success in patients with MSCAC, which include lesion length, MLD, TIMI, CTO, RVD, and lesion with diffuse range. Lesion length was the most important predictive factor in CAG for immediate successful PCI in patients with MSCAC with a SHAP value of 1.65. Figure 3C indicates whether each feature for a given observation is considered high (represented in yellow) or low (represented in blue-green) based on its corresponding SHAP value.

### ML Performance Testing and External Validation

A total of 1437 patients with MSCAC who underwent PCI were extracted from the 2013 database for use as a testing dataset to validate the predictive accuracy of the selected XGBoost model. Table S1 in Multimedia Appendix 1 presents the baseline characteristics of these patients. Among them, a total of 147 (10.2%) patients experienced failure during the PCI procedure. Collectively, the simplified XGBoost model, constructed using the top 6 features, demonstrated robust predictive performance, achieving AUC 0.972, AP 0.962, *F*_1_-score 0.940, and G-mean 0.930. According to the findings from the DCA, the XGBoost model exhibited favorable clinical utility within the testing set, as depicted in Figure S5 in Multimedia Appendix 2.

In the external validation, 204 patients with MSCAC met the inclusion criteria, among whom 15 (7.4%) experienced PCI failure. The simplified XGBoost model demonstrated relatively good performance in this cohort, with an AUC of 0.810, an AP of 0.957, an *F*_1_-score of 0.892, and a G-mean of 0.798. DCA further indicated its potential clinical utility (Figure S6 in Multimedia Appendix 2).

### Relationship Between CAG Characteristics and PCI Failure

The lesion length, MLD, and RVD were found to have a nonlinear relationship with the probability of PCI failure with the cutoff value of 30, 0.3, and 3.0 mm, respectively (Figure S7 in Multimedia Appendix 2). When the value of MLD was lower than 0.3, the risk of PCI failure in patients with MSCAC increased dramatically with the decrease of MLD. Univariable and multivariable LR showed that lesion length was positively associated with PCI failure (adjusted odds ratio [OR] 2.317, 95% CI 1.740‐3.086 as categorical) and various reasons (post-TIMI 0 or 1, RS ≥50%, and perforation risk; Table S6 in Multimedia Appendix 1). MLD was significantly associated with PCI failure (adjusted OR 5.564, 95% CI 4.174‐7.417 as categorical, <0.3 mm regarded as events) and post-TIMI 0 or 1, RS ≥50%, dissection, perforation risk, and in-hospital MACE (Table 3). The same correlation was found in RVD and PCI failure (adjusted OR 1.924, 95% CI 1.469‐2.519 as categorical, <3.0 mm regarded as events) and various reasons (Table S7 in Multimedia Appendix 1).

**Table 3.** The relationship between percutaneous coronary intervention (PCI) failure and minimal lumen diameter (MLD)^a^.

	Model 1		Model 2	
	OR^b^ (95% CI)	*P *value	Adjusted OR (95% CI)	*P *value
Continue				
PCI failure	0.007 (0.003‐0.014)	<.001	0.007 (0.007‐0.141)	<.001
TIMI^c^ 0 or 1	0.001 (0.000‐0.001)	<.001	0.001 (0.000‐0.001)	<.001
RS^d^ >50%	0.002 (0.001‐0.004)	<.001	0.002 (0.001‐0.004)	<.001
Dissection	0.035 (0.0007‐0.179)	<.001	0.036 (0.007‐0.181)	<.001
Thrombosis	0.242 (0.013‐4.343)	.34	0.236 (0.013‐4.284)	.33
Perforation	0.039 (0.012‐0.129)	<.001	0.038 (0.012‐0.123)	<.001
In-hospital MACE^e^	0.049 (0.009‐0.259)	<.001	0.042 (0.007‐0.226)	<.001
Category				
PCI Failure	6.327 (4.477‐8.940)	<.001	6.393 (4.521‐9.039)	<.001
TIMI 0 or 1	64.393 (20.581‐201.470)	<.001	64.895 (20.738‐203.077)	<.001
RS >50%	9.601 (6.181‐14.937)	<.001	9.704 (6.238‐15.094)	<.001
Dissection	4.631 (1.952‐10.986)	.001	4.633 (1.952‐10.994)	.001
Thrombosis	1.441 (0.359‐5.772)	.61	1.441 (0.359‐5.778)	.61
Perforation	3.295 (1.875‐5.794)	<.001	3.313 (1.884‐5.827)	<.001
In-hospital MACE	2.818 (1.292‐6.151)	<.001	2.858 (1.308‐6.245)	.008

a Adjust for sex, age, BMI, dyslipidemia, and smoking. When MLD was analyzed as a category variable, MLD ≤0.3 mm was defined as an event (1).

b OR: odds ratio.

c TIMI: thrombolysis in myocardial infarction.

d RS: residual stenosis.

e MACE: major adverse cardiovascular event.

### Validation in Patients With No or Mild Coronary Calcification

To further assess the performance of the model, it was validated in patients with no or mild CAC. A total of 17,998 patients were included in the study, with specific inclusion and exclusion criteria and baseline data detailed in Table S2 in Multimedia Appendix 1 and Figure S1 in Multimedia Appendix 2. Among these patients, 774 experienced PCI failure, representing approximately 4.3%. The simplified XGBoost model using the 6 most important variables showed significantly reduced predictive performance in this population compared to the patients with MSCAC (AUC 0.843; AP 0.357). DCA indicated that the model had limited clinical predictive value in patients with no or mild CAC (Figure S8 in Multimedia Appendix 2).

To identify the CAG characteristics associated with PCI failure in patients with no or mild CAC, the SHAP algorithm was applied. The 6 most valuable features for predicting PCI failure were MLD, lesion length, TIMI flow, RVD, CTO, and degree of calcification. The importance of the top 5 key features in patients with no or mild calcification has changed compared to patients with MSCAC, but the types have not changed. For patients with MSCAC, diffuse lesions ranked sixth, whereas in those with no or mild calcification, the sixth feature was the degree of calcification. Figure S9 in Multimedia Appendix 2 illustrates the importance of each variable based on the SHAP algorithm.

### PCI Procedures and Success

**Figure 4.**
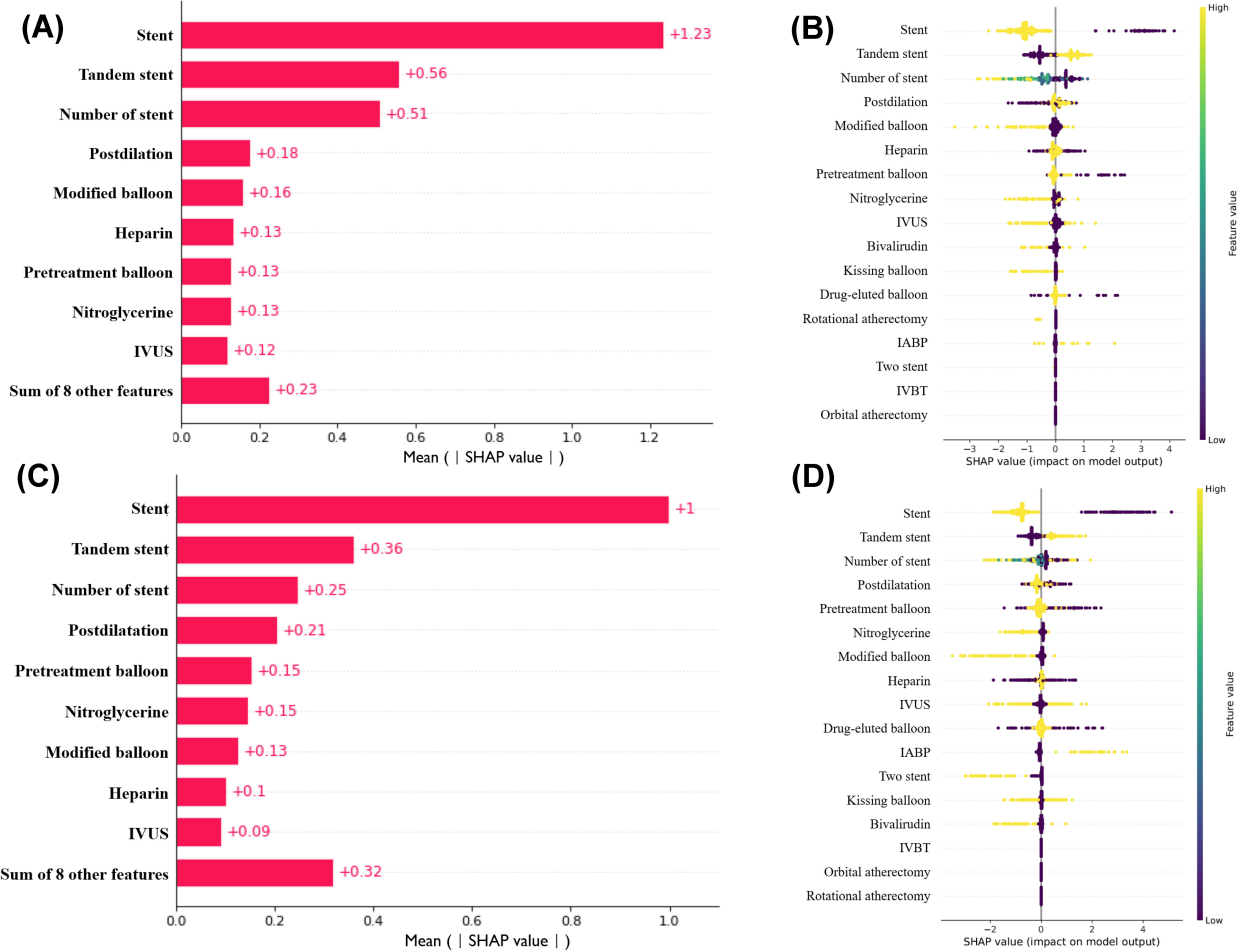
The importance of various predictors of PCI treatment in various patients with CAC. (A and B) Rank the importance of the significant PCI treatments to predict the PCI success rate in patients with moderate to severe coronary artery calcification. (C and D) Rank the importance of the significant PCI treatments to predict the PCI success rate in no or patients with mild CAC. CAC: coronary artery calcification; IABP: intra-aortic balloon pump; IVBT: intravascular brachytherapy; IVUS: intravascular ultrasound; PCI: percutaneous coronary intervention.

Building upon the SHAP algorithm of the XGBoost model, we explored the significant predictive value of different PCI procedures and their correlation with PCI success in patients with both MSCAC and no or mild CAC. In the MSCAC cohort, the 5 most important PCI procedures predicting success were stent implantation, the use of the tandem stenting technique, the number of stents implanted, postdilation, and the use of modified balloons (including cutting balloons and scoring balloons). In contrast, for patients with no or mild CAC, the 5 most important PCI procedures predicting success included stent implantation, the use of the tandem stenting technique, the number of stents implanted, postdilation, and the use of balloon predilation (Figure 4).

### Sensitivity Analysis

We further investigated the association between coronary angiographic characteristics and PCI success in both patients with MSCAC and ACS and patients with MSCAC and chronic coronary syndrome (CCS). The results from the SHAP analysis indicated that the 5 most important coronary angiographic features predicting PCI success were the same for patients with both ACS and CCS, with consistent importance rankings: lesion length, MLD, TIMI flow, RVD, and CTO. The simplified XGBoost algorithm maintained high predictive value in patients with both ACS and CCS (AUC 0.992 and 0.974, respectively; Figure S10 in Multimedia Appendix 2).

## Discussion

### Principal Findings

This study explored the coronary angiographic characteristics and PCI procedures that have the highest predictive value for PCI success rates in patients with MSCAC using ML approaches. By comparing the performance of various ML algorithms, we ultimately selected the XGBoost algorithm, which demonstrated the highest predictive efficacy for assessing PCI success rates. Using the SHAP algorithm, we identified 6 critical coronary artery features—lesion length, MLD, TIMI flow, CTO, RVD, and diffuse lesion—as the most significant predictors of PCI success in patients with MSCAC. A simplified XGBoost model constructed with these 6 variables exhibited robust predictive performance and clinical value in the validation cohort. Additionally, we determined the cutoff values for lesion length, MLD, and RVD. Furthermore, by comparing the results with those from patients with no or mild CAC, we found that the use of modified balloons (including cutting and scoring balloons) for the preparation of calcified lesions was beneficial for PCI success in patients with MSCAC.

The success rate of interventional procedures in patients with MSCAC is lower compared to patients with no or mild calcification [28]. In this study, the PCI success rate for patients with MSCAC was 92.4% (n=3023), while the success rate for patients with no or mild CAC was 95.5%, which was significantly higher than that of the MSCAC group. These findings are consistent with previous research results. This discrepancy may be attributed to the presence of severe calcified lesions in MSCAC, which can complicate PCI treatment [19]. Significant calcification increases the risk of inadequate stent expansion and stent fracture after the procedure, thereby contributing to intervention failure [29]. Furthermore, the coronary artery anatomy of patients with MSCAC is often more complex than that of patients with no or mild calcification, leading to a higher probability of intricate lesions such as CTO and angulated lesions [30]. This complexity raises the technical demands on physicians performing PCI. Patients with more severe calcification have a significantly reduced success rate for PCI, which may partly explain their poorer prognoses [19,29]. Severe calcified lesions can lead to various causes of PCI failure, including difficulties in wire passage, inadequate balloon expansion, and unsuccessful stent placements [18,31].

Previous studies have demonstrated that patients with MSCAC and ACS exhibit a 44% higher likelihood of treatment failure from target lesions than those with less severe calcification [32]. The difficulty of performing emergency PCI in patients with ACS is greater than that in patients with CCS, which may have a potential impact on PCI success rates. Therefore, we conducted a sensitivity analysis in patients with MSCAC to identify the key coronary angiographic features associated with PCI failure in both ACS and CCS populations. The top 5 coronary angiographic features were consistent between the 2 groups, suggesting that ACS does not play a significant role in predicting PCI success rates in patients with MSCAC. This highlights the need for further targeted research to explore this issue.

This study identified lesion length, MLD, TIMI, CTO, RVD, and diffuse range lesion as the 6 most significant vascular characteristics predictive of PCI success in patients with MSCAC. The SHAP value for lesion length was the highest, at 1.65, indicating that clinicians should pay closer attention to lesion length; for instance, a lesion length exceeding 30 mm suggests a higher likelihood of PCI failure. Longer lesion lengths indicate a greater complexity in lesion management, with increasing unknown variables necessitating consideration of more complex intervention strategies, such as stent thrombosis and overlap, which undoubtedly amplifies the challenge of PCI [33,34]. A smaller MLD correlates with increased difficulty in wire passage; prior research has linked MLD to poor outcomes following intervention in patients with ACS [35]. Once CTO occurs in patients with MSCAC, the difficulty and risks associated with PCI become substantially heightened, significantly decreasing the likelihood of procedural success [36]. Furthermore, the choice of PCI strategy in cases of diffuse lesions presents additional challenges; severe calcification combined with diffuse range further complicates the procedure and may reduce the success rate [37]. The coronary angiographic metrics used in this study, such as MLD and RVD, are clinician-interpreted CAG features rather than derived from quantitative CAG measurements, which may introduce some degree of bias. However, these CAG features were independently measured by 2 experienced operators, and in cases of significant discrepancies, a third expert was consulted to ensure accuracy.

For patients with MSCAC, the smooth execution of the PCI procedure remains a critical factor influencing prognosis [38]. This study further explored the important PCI procedures related to PCI success in patients with MSCAC compared to those with no or mild CAC. The results from the SHAP algorithm indicated that the presence of stent implantation, the use of tandem stenting techniques, the number of stents implanted, and postdilation were common PCI procedures influencing success in both patient groups. Notably, the use of modified balloons (including cutting balloons and scoring balloons) had a specific impact in patients with MSCAC, with a SHAP value of 0.16, compared to a SHAP value of 0.13 in patients with no or mild CAC. Cutting balloons (Wolverine Cutting Balloon, Boston Scientific) are noncompliant balloons with 3 or 4 microblades arranged longitudinally on their surface [18]. Previous studies have suggested that the Wolverine cutting balloon is effective in treating significant CAC, particularly in cases of inadequate stent expansion and in circumferentially calcified coronary lesions [39,40]. Scoring balloons are semicompliant (AngioSculpt, Philips; NSE Alpha, Braun) or noncompliant (ScoreFlex noncompliant, OrbusNeich) balloons with scoring elements on their surface. A randomized trial comparing high-pressure balloons with scoring balloons for the preparation of calcified coronary lesions showed comparable stent expansion on intravascular imaging [41]. This study supports the positive role of modified balloons in PCI treatment for patients with MSCAC.

Rotational atherectomy is considered a beneficial tool for facilitating balloon and stent delivery and achieving adequate stent expansion in calcified coronary lesions, particularly when balloon-based lesion preparation strategies fail [42]. However, it was not included among the top 5 procedures beneficial for PCI success in this study. Nonetheless, its significant role in patients with CAC cannot be overlooked, as its overall application in the MSCAC population in this study was 2.4%, indicating that this technique was not yet widely implemented in China at that time. Intravascular imaging techniques, such as intravascular ultrasound (IVUS) and optical coherence tomography (OCT), play a crucial role in PCI procedures for patients with CAC. IVUS and OCT can quantify calcification by assessing the arc size and length of calcified segments, providing valuable information about coronary calcification, and guiding PCI in various complex cases [18,43]. Although IVUS was not listed among the top 5 beneficial procedures for PCI success in this study, it was used in 11.6% of patients who had successful PCI compared to only 9.7% of those who failed, demonstrating a statistically significant difference. This suggests the guiding value of intravascular imaging in patients with CAC; however, in our study, the use of IVUS and OCT was limited, possibly due to economic constraints. In summary, the relationship between different PCI strategies for CAC, PCI success rates, and outcomes warrants further exploration through prospective cohort studies.

Noninvasive multimodal imaging plays a crucial role in PCI for patients with complex coronary lesions [44,45]. Coronary computed tomography angiography has been validated as an accurate noninvasive method for detecting coronary artery stenosis, allowing for the assessment and quantification of calcium distribution and providing a roadmap for all coronary arteries [46]. Its information has low spatial resolution and systematically overestimates the volume of calcified plaques. However, coronary computed tomography angiography has not yet been fully used in clinical practice. Additionally, echocardiography, cardiac magnetic resonance imaging, and nuclear cardiac imaging play important roles in assessing myocardial viability, measuring left ventricular function, and simultaneously evaluating myocardial ischemia [44]. In patients with severe CAC, the complexity of coronary lesions necessitates the integration of assessments of myocardial viability, left ventricular function, and individual risk status and the detection of inducible ischemia, as these are key pieces of information for shared treatment decisions and intervention strategy planning to enhance PCI success rates.

Artificial intelligence has been extensively applied in the field of cardiovascular medicine. XGBoost, a scalable parallel boosting tree algorithm, is also an iterative upgrade of the GBDT. XGBoost is currently recognized as an effective open-source boosting tree toolkit, characterized by its efficiency, flexibility, and compactness, and has been widely used in data mining and recommendation systems [47,48]. It effectively captures complex nonlinear relationships and interactions among multiple imaging features in patients with CAC, which are often inadequately expressed by traditional linear or simple models. Additionally, XGBoost exhibits strong noise resistance and adaptability to high-dimensional, heterogeneous clinical data, thereby enhancing the precision of predicting PCI failure risk. Consequently, this model demonstrates superior performance in these clinical settings.

In our research, we constructed a simplified XGBoost model based on the 6 most important vascular features, which achieved excellent validation results in external assessments. The application of this model should be integrated into CAG systems to provide timely assistance and guidance to clinicians performing PCI on patients with MSCAC and alleviate operators’ concerns regarding the treatment of these patients with complex coronary disease, similar to angiographic quantitative flow ratio calculation systems [49]. For example, if a patient with MSCAC is predicted by this model to have a low success rate for PCI, the operator can communicate the increased technical difficulty and risks with the patient and their family. For patients with financial difficulties who cannot afford treatment and patients unwilling to afford the risks of PCI, a more comprehensive PCI treatment will be reconsidered or avoided. Conversely, for patients who are able to afford the procedure and accept associated risks, a cautious approach to PCI can be continued. Therefore, this model can aid patients in balancing economic investment with health benefits, help cardiologists mitigate the risk of PCI failure, optimize resource allocation, and assist in clinical decision-making for PCI in patients with MSCAC.

### Limitations

First, the cohorts included in this study are single-center, nonrandomized investigations, which may introduce selection bias and uneven baseline characteristics. This study exclusively involved individuals from China, which may limit the international applicability of our findings. Future studies should explore the model’s applicability in other populations. This limitation also restricts the generalizability of the ML model for practical application in multicenter settings, necessitating further validation with multicenter data. Second, the vascular characteristics studied were interpreted by clinicians based on their own experience in assessing CAG images. Although CAG demonstrates a specificity of up to 89% for diagnosing calcified lesions, with a specificity of 98% for severe calcifications, the definition of CAC is qualitative, which may have introduced selection bias. Deep learning algorithms can recognize various characteristic features in CAG, such as convolutional neural networks. Therefore, further research using algorithms based on deep learning for the identification of CAG features is warranted. Finally, the latest PCI treatment measures for CAC, such as rotational atherectomy, orbital atherectomy, excimer laser coronary atherectomy, and intravascular lithotripsy, were not routinely performed in this study, limiting our ability to further investigate their predictive value for PCI success in patients with MSCAC.

### Conclusions

The interpretable XGBoost models with the best performance in predicting PCI immediate procedure success for patients with MSCAC were established and validated. Six important CAG predictors, including lesion length, MLD, TIMI, CTO, RVD, and diffuse lesion, and a PCI treatment (modified balloon) were explored. ML models can precisely predict the success rate of PCI in patients with MSCAC. This capability assists clinicians in identifying challenging cases, enabling cardiologists to allocate their efforts more effectively toward delivering safer and more efficient treatments.

## Supplementary material

10.2196/70943 Multimedia Appendix 1 Supplementary tables (Tables S1-S7).

10.2196/70943 Multimedia Appendix 2 Supplementary figures (Figures S1-S10).

## Abbreviations

ACS: acute coronary syndrome
AP: average precision
AUC: area under the receiver operator characteristic curve
CAC: coronary artery calcification
CAD: coronary artery disease
CAG: coronary angiography
CCS: chronic coronary syndrome
CTO: chronic total occlusion
DCA: decision curve analysis
GBDT: gradient boosting decision tree machine
IVUS: intravascular ultrasound
KNN: k-nearest neighbor
LR: logistic regression
MACE: major adverse cardiovascular event
ML: machine learning
MLD: minimal lumen diameter
MSCAC: moderate to severe coronary artery calcification
OCT: optical coherence tomography
OR: odds ratio
PCI: percutaneous coronary intervention
RS: residual stenosis
RVD: reference vessel diameter
SHAP: Shapley Additive Explanations
SMOTE: synthetic minority oversampling technique
TIMI: thrombolysis in myocardial infarction
XGBoost: Extreme Gradient Boosting
