# Correction: Prediction of Percutaneous Coronary Intervention Success in Patients With Moderate to Severe Coronary Artery Calcification Using Machine Learning Based on Coronary Angiography: Prospective Cohort Study

**DOI:** 10.2196/80773

**Published:** 2025-07-30

**Authors:** Zixiang Ye, Zhangyu Lin, Enmin Xie, Chenxi Song, Rui Zhang, Hao-Yu Wang, Shanshan Shi, Lei Feng, Kefei Dou

**Affiliations:** 1 Department of Cardiology, Fuwai Hospital, Chinese Academy of Medical Sciences & Peking Union Medical College, No 167 North Lishi Road, Xicheng District, Beijing, 100037, China, 86 88398866; 2 State Key Laboratory of Cardiovascular Disease, Beijing, China; 3 Cardiometabolic Medicine Center, Fuwai Hospital, Chinese Academy of Medical Sciences & Peking Union Medical College, Beijing, China

**Keywords:** extreme gradient boosting, artificial intelligence, coronary angiography, coronary artery calcification, percutaneous coronary intervention

In “Prediction of Percutaneous Coronary Intervention Success in Patients With Moderate to Severe Coronary Artery Calcification Using Machine Learning Based on Coronary Angiography: Prospective Cohort Study” (J Med Internet Res 2025;27:e70943), the authors noted two errors.

The corresponding author’s name was corrected to read as “Kefei Dou.”

In addition, the grant number for the Beijing Natural Science Foundation has been corrected in the Acknowledgements section to the following: “24L60308.”

The correction will appear in the online version of the paper on the JMIR Publications website, together with the publication of this correction notice. Because this was made after submission to PubMed, PubMed Central, and other full-text repositories, the corrected article has also been resubmitted to those repositories.

