# Supplementary material for: Prediction of Percutaneous Coronary Intervention Success in Patients With Moderate to Severe Coronary Artery Calcification Using Machine Learning Based on Coronary Angiography: Prospective Cohort Study
Source: J Med Internet Res. 2025 Jul 11;27:e70943. doi: 10.2196/70943 (PMC12274018; doi:10.2196/70943)
Supplement: Multimedia Appendix 1 [file jmir-v27-e70943-s001.docx]

## Supplementary Tables

**Table S1** Baseline characteristic of external test set including PCI patients from Jan. 2013 to Dec. 2013.

**Table S2** baseline characteristics of no or mild CAC patients from 2017 to 2018.

**Table S3** baseline characteristic of external validation set including MSCAC patients underwent PCI in a general hospital from Jan. 2021 to Dec. 2021.

**Table S4** PCI failure and reasons in both development set and validation set.

**Table S5** Important Parameters of XGBoost after the hyperparameter optimization process.

**Table S6** the relationship between PCI failure and Lesion length.

**Table S7** the relationship between PCI failure and RVD.

| **Table S1 Baseline characteristic of testing set including PCI patients in 2013** | | | | | |
| --- | --- | --- | --- | --- | --- |
|  | **level** | **Overall** | **PCI Success** | **PCI Failure** | **p** |
| n |  | 1437 | 1290 | 147 |  |
| Male (%) |  | 1054 (73.3) | 945 (73.3) | 109 (74.1) | 0.894 |
| Age |  | 59.83 (10.62) | 59.88 (10.58) | 59.35 (11.02) | 0.564 |
| Pre Syntax |  | 14.51 (8.31) | 14.27 (8.24) | 16.56 (8.70) | 0.002 |
| BMI |  | 25.80 (3.30) | 25.77 (3.26) | 26.11 (3.59) | 0.227 |
| LVEF,% |  | 62.90 (7.30) | 63.02 (7.09) | 61.87 (8.89) | 0.072 |
| T2DM (%) |  | 464 (32.3) | 419 (32.5) | 45 (30.6) | 0.714 |
| Dyslipidemia (%) |  | 937 (65.2) | 838 (65.0) | 99 (67.3) | 0.628 |
| Family History CAD (%) |  | 1428 (99.4) | 1283 (99.5) | 145 (98.6) | 0.523 |
| Smoking (%) |  | 768 (53.4) | 696 (54.0) | 72 (49.0) | 0.29 |
| Anomalous origin coronary (%) |  | 20 (1.4) | 17 (1.3) | 3 (2.0) | 0.736 |
| Irregular (%) |  | 10 (0.7) | 8 (0.6) | 2 (1.4) | 0.617 |
| RCA (%) |  | 859 (59.8) | 766 (59.4) | 93 (63.3) | 0.411 |
| LM (%) |  | 100 (7.0) | 92 (7.1) | 8 (5.4) | 0.554 |
| LAD (%) |  | 1033 (71.9) | 938 (72.7) | 95 (64.6) | 0.049 |
| LCX (%) |  | 974 (67.8) | 875 (67.8) | 99 (67.3) | 0.98 |
| Triple vessel (%) |  | 601 (41.8) | 539 (41.8) | 62 (42.2) | 0.997 |
| TVD.LM (%) |  | 75 (5.2) | 68 (5.3) | 7 (4.8) | 0.946 |
| Lesion length, mm |  | 34.61 (23.28) | 34.10 (19.89) | 39.07 (42.61) | <0.001 |
| DS,% |  | 89.78 (8.29) | 89.26 (8.11) | 94.35 (8.49) | <0.001 |
| MLD, mm |  | 0.32 (0.26) | 0.33 (0.26) | 0.16 (0.25) | <0.001 |
| TIMI (%) | 0 | 331 (23.0) | 251 (19.5) | 80 (54.4) | <0.001 |
|  | 1 | 49 (3.4) | 42 (3.3) | 7 (4.8) |  |
|  | 2 | 209 (14.5) | 198 (15.3) | 11 (7.5) |  |
|  | 3 | 848 (59.0) | 799 (61.9) | 49 (33.3) |  |
| B2_C_lesion (%) |  | 1178 (82.0) | 1051 (81.5) | 127 (86.4) | 0.175 |
| Diffused Range (%) |  | 1059 (73.7) | 944 (73.2) | 115 (78.2) | 0.223 |
| Extremely Tortuosity (%) |  | 58 (4.0) | 43 (3.3) | 15 (10.2) | <0.001 |
| Angulated (%) |  | 606 (42.8) | 537 (42.2) | 69 (48.9) | 0.146 |
| Calcified degree (%) | Moderate | 1175 (81.8) | 1071 (83.0) | 104 (70.7) | <0.001 |
|  | Heavily | 262 (18.2) | 219 (17.0) | 43 (29.3) |  |
| TO (%) |  | 338 (23.5) | 257 (19.9) | 81 (55.1) | <0.001 |
| CTO (%) |  | 181 (12.6) | 120 (9.3) | 61 (41.5) | <0.001 |
| Ostial (%) |  | 225 (15.7) | 199 (15.4) | 26 (17.7) | 0.552 |
| Bifurcation (%) |  | 260 (18.1) | 246 (19.1) | 14 (9.5) | 0.006 |
| Pre Thrombosis (%) |  | 40 (2.8) | 30 (2.3) | 10 (6.8) | 0.004 |
|  |  |  |  |  |  |
| RVD, Reference vessel diameter; T2DM, type2 diabetes mellitus; LVEF, left ventricular ejection fraction; CAD, coronary artery disease; MLD, minimal lumen diameter; DS, degree of stenosis; TIMI, Thrombolysis In Myocardial Infarction; LM, left main coronary artery; RCA, right coronary artery; LAD, left anterior descending branch; LCX, left circumflex branch; TO, total occlusion; CTO, chronic total occlusion; BMI, Body Mass Index. | | | | | |

| **Table S2 baseline characteristic of no or mild CAC patients from 2017 to 2018** | | | | | |
| --- | --- | --- | --- | --- | --- |
|  |  | **Overall** | **PCI Success** | **PCI Failure** | **p** |
| n |  | 17998 | 17224 | 774 |  |
| Sex (%) |  | 13644 (75.8) | 13007 (75.5) | 637 (82.3) | <0.001 |
| Age, y |  | 59.60 [52.30, 65.90] | 59.60 [52.30, 65.90] | 59.50 [51.60, 66.10] | 0.581 |
| BMI |  | 25.73 [23.83, 27.77] | 25.71 [23.80, 27.76] | 26.42 [24.22, 28.41] | <0.001 |
| ACS (%) |  | 11645 (64.7) | 11180 (64.9) | 465 (60.1) | 0.007 |
| T2DM (%) |  | 5608 (31.2) | 5369 (31.2) | 239 (30.9) | 0.895 |
| Dyslipidemia (%) |  | 13323 (74.0) | 12739 (74.0) | 584 (75.5) | 0.377 |
| Family History CAD (%) |  | 1928 (10.7) | 1838 (10.7) | 90 (11.6) | 0.434 |
| Smoking (%) |  | 11697 (65.0) | 11129 (64.6) | 568 (73.4) | <0.001 |
| No calcified (%) |  | 11600 (64.5) | 11121 (64.6) | 479 (61.9) | 0.137 |
| Mild calcified (%) |  | 6398 (35.5) | 6103 (35.4) | 295 (38.1) | 0.137 |
| LM (%) |  | 734 (4.1) | 722 (4.2) | 12 (1.6) | <0.001 |
| LAD (%) |  | 8236 (45.8) | 7954 (46.2) | 282 (36.4) | <0.001 |
| LCX (%) |  | 2900 (16.1) | 2795 (16.2) | 105 (13.6) | 0.055 |
| RCA (%) |  | 5919 (32.9) | 5560 (32.3) | 359 (46.4) | <0.001 |
| Pre-Syntax |  | 11.00 [7.00, 18.50] | 11.00 [7.00, 18.00] | 18.25 [10.00, 24.50] | <0.001 |
| Triple vessel (%) |  | 8091 (45.0) | 7635 (44.3) | 456 (58.9) | <0.001 |
| LM disease (%) |  | 1468 (8.2) | 1422 (8.3) | 46 (5.9) | 0.026 |
| MLD, mm |  | 0.37 (0.42) | 0.38 (0.42) | 0.07 (0.26) | <0.001 |
| RVD, mm |  | 3.16 (2.00) | 3.16 (2.02) | 3.06 (1.33) | 0.158 |
| DS, % |  | 88.45 (9.31) | 88.03 (9.17) | 97.86 (7.10) | <0.001 |
| Pre TIMI (%) | 0 | 3033 (16.9) | 2399 (13.9) | 634 (81.9) | <0.001 |
|  | 1 | 664 (3.7) | 640 (3.7) | 24 (3.1) |  |
|  | 2 | 1748 (9.7) | 1736 (10.1) | 12 (1.6) |  |
|  | 3 | 12553 (69.7) | 12449 (72.3) | 104 (13.4) |  |
| B2_C_lesion (%) |  | 12994 (72.2) | 12268 (71.2) | 726 (93.8) | <0.001 |
| Diffused Range (%) |  | 10613 (59.0) | 10043 (58.3) | 570 (73.6) | <0.001 |
| Lesion length |  | 25.00 [16.00, 37.00] | 25.00 [16.00, 36.00] | 30.00 [20.00, 50.00] | <0.001 |
| Concentric (%) |  | 2944 (16.4) | 2788 (16.2) | 156 (20.2) | 0.004 |
| Extremely Tortuosity (%) |  | 339 (1.9) | 300 (1.7) | 39 (5.0) | <0.001 |
| Angulated (%) |  | 5593 (31.1) | 5303 (30.8) | 290 (37.5) | <0.001 |
| Irregular (%) |  | 13234 (73.5) | 12589 (73.1) | 645 (83.3) | <0.001 |
| CTO (%) |  | 1662 (9.2) | 1220 (7.1) | 442 (57.1) | <0.001 |
| Ostial (%) |  | 2059 (11.4) | 1932 (11.2) | 127 (16.4) | <0.001 |
| Bifurcation (%) |  | 3598 (20.0) | 3497 (20.3) | 101 (13.0) | <0.001 |
| Pre-Thrombosis (%) |  | 836 (4.6) | 745 (4.3) | 91 (11.8) | <0.001 |
| Modified Balloon (%) |  | 936 (5.2) | 922 (5.4) | 14 (1.8) | <0.001 |
| Rotational Atherectomy (%) |  | 22 (0.1) | 20 (0.1) | 2 (0.3) | 0.56 |
| Stent (%) |  | 16724 (92.9) | 16478 (95.7) | 246 (31.8) | <0.001 |
| Stent. num |  | 1.00 [1.00, 2.00] | 1.00 [1.00, 2.00] | 2.00 [1.00, 2.00] | 0.167 |
| Post Dilatation (%) |  | 13650 (75.8) | 13209 (76.7) | 441 (57.0) | <0.001 |
| Tandem stent (%) |  | 5831 (32.4) | 5469 (31.8) | 362 (46.8) | <0.001 |
| Balloon (%) |  | 17516 (97.3) | 16970 (98.5) | 546 (70.5) | <0.001 |
| IVUS (%) |  | 1661 (9.2) | 1588 (9.2) | 73 (9.4) | 0.892 |
| Kissing Balloon (%) |  | 1102 (6.1) | 1090 (6.3) | 12 (1.6) | <0.001 |
| Two Stent (%) |  | 430 (2.4) | 425 (2.5) | 5 (0.6) | 0.002 |
| IABP (%) |  | 146 (0.8) | 115 (0.7) | 31 (4.0) | <0.001 |
| Heparin (%) |  | 14932 (83.0) | 14318 (83.1) | 614 (79.3) | 0.007 |
| Bivalirudin (%) |  | 641 (3.6) | 614 (3.6) | 27 (3.5) | 0.99 |
| Nitroglycerine (%) |  | 2594 (14.4) | 2543 (14.8) | 51 (6.6) | <0.001 |
| Pretreatment balloon (%) |  | 16888 (93.8) | 16293 (94.6) | 595 (76.9) | <0.001 |
|  |  |  |  |  |  |
| RVD, Reference vessel diameter; T2DM, type2 diabetes mellitus; LVEF, left ventricular ejection fraction; CAD, coronary artery disease; MLD, minimal lumen diameter; DS, degree of stenosis; TIMI, Thrombolysis In Myocardial Infarction; LM, left main coronary artery; RCA, right coronary artery; LAD, left anterior descending branch; LCX, left circumflex branch; TO, total occlusion; CTO, chronic total occlusion; BMI, Body Mass Index. | | | | | |

| **Table S3 baseline characteristic of external validation set including MSCAC patients underwent PCI in a general hospital from Jan. 2021 to Dec. 2021.** | | | | | |
| --- | --- | --- | --- | --- | --- |
|  |  | Overall | PCI Success | PCI Failure | p |
| n |  | 204 | 189 | 15 |  |
| Male (%) |  | 151 (74.0) | 142 (75.1) | 9 (60.0) | 0.327 |
| Age, y |  | 62.60 [55.68, 70.62] | 62.40 [55.60, 69.90] | 63.70 [60.55, 73.75] | 0.426 |
| BMI |  | 25.40 [23.34, 27.62] | 25.35 [23.18, 27.61] | 25.78 [23.96, 27.73] | 0.733 |
| LAD (%) |  | 73 (35.8) | 72 (38.1) | 1 (6.7) | 0.03 |
| LCX (%) |  | 35 (17.2) | 34 (18.0) | 1 (6.7) | 0.445 |
| RCA (%) |  | 87 (42.6) | 74 (39.2) | 13 (86.7) | 0.001 |
| Pre-Syntax |  | 11.00 [7.00, 17.25] | 11.00 [7.00, 17.00] | 15.00 [10.50, 28.25] | 0.022 |
| MLD, mm |  | 0.30 [0.09, 0.50] | 0.30 [0.15, 0.50] | 0.00 [0.00, 0.00] | <0.001 |
| RVD, mm |  | 3.00 [2.50, 3.50] | 3.00 [2.50, 3.50] | 3.00 [3.00, 3.50] | 0.261 |
| DS, % |  | 90.00 [80.00, 96.00] | 90.00 [80.00, 95.00] | 100.00 [100.00, 100.00] | <0.001 |
| Pre TIMI (%) | 0 | 46 (22.5) | 34 (18.0) | 12 (80.0) | <0.001 |
|  | 1 | 5 (2.5) | 5 (2.6) | 0 (0.0) |  |
|  | 2 | 21 (10.3) | 20 (10.6) | 1 (6.7) |  |
|  | 3 | 132 (64.7) | 130 (68.8) | 2 (13.3) |  |
| Diffused Range (%) | | 120 (58.8) | 110 (58.2) | 10 (66.7) | 0.012 |
| Lesion length |  | 25.50 [15.00, 40.00] | 25.00 [15.00, 40.00] | 30.00 [20.00, 37.50] | 0.022 |
| Concentric (%) |  | 21 (10.3) | 20 (10.6) | 1 (6.7) | 0.969 |
| Extremely Tortuosity (%) | | 16 (7.8) | 14 (7.4) | 2 (13.3) | 0.747 |
| CTO (%) |  | 30 (14.7) | 20 (10.6) | 10 (66.7) | <0.001 |
| Pre Thrombosis (%) | | 17 (8.3) | 15 (7.9) | 2 (13.3) | 0.808 |
| RVD, Reference vessel diameter; CAD, coronary artery disease; MLD, minimal lumen diameter; DS, degree of stenosis; TIMI, Thrombolysis In Myocardial Infarction; RCA, right coronary artery; LAD, left anterior descending branch; LCX, left circumflex branch; CTO, chronic total occlusion; BMI, Body Mass Index. | | | | | |

| **Table S4 PCI failure and reasons in both development set and validation set** | | | |
| --- | --- | --- | --- |
|  | **Development set (2017-2018)** | **External validation set (2013)** | |
| PCI Failure | 248(7.6%) | 147(10.2%) |  |
| post RS>50% | 196(6.0%) | 105(7.3%) |  |
| post TIMI 0 or 1 | 191(5.8%) | 65(4.5%) |  |
| post Dissection | 44(1.3%) | 36(2.5%) |  |
| post Thrombosis | 9(0.3%) | 4(0.3%) |  |
| post perforation | 82(2.5%) | 10(0.8%) |  |
| in-hospital MACE | 39(1.2%) | 21(1.5%) |  |
|  |  |  |  |
| PCI, percutaneous coronary intervention; RS, residual stenosis; TIMI, Thrombolysis In Myocardial Infarction; MACE, major adverse cardiovascular events. | | | |

| **Table S5 Important Parameters of XGBoost after hyperparameter optimization process** | |
| --- | --- |
| **Parameters** | **value** |
| Learning rate | 0.05 |
| N estimators | 2800 |
| Max depth | 5 |
| Min child weight | 1 |
| gamma | 0.21 |
| subsample | 0.8 |
| Colsample bytree | 0.75 |
| objective | binary:logistic |

| **Table S6 the relationship between PCI failure and Lesion length.** | | | | |
| --- | --- | --- | --- | --- |
|  | **Model1** |  | **Model2** |  |
| **continue** | **OR (95%CI)** | **P** | **adjusted OR (95%CI)** | **P** |
| PCI Failure | 1.432(1.005-1.729) | 0.005 | 1.313(1.007-1.628) | 0.006 |
| TIMI 0 or 1 | 1.453(1.243-1.698) | <0.001 | 1.401(1.233-1.668) | <0.001 |
| RS >50% | 1.201(0.996-1.003) | 0.123 | 1.118(1.003-1.203) | 0.026 |
| Dissection | 1.000(0.998-1.004) | 0.644 | 1.000(0.998-1.003) | 0.623 |
| Thrombosis | 1.071(0.736-1.560) | 0.718 | 1.047(0.715-1.533) | 0.813 |
| Perforation | 1.035(0.978-1.072) | 0.084 | 1.054(1.004-1.102) | 0.046 |
| In hospital MACE | 1.000(0.998-1.003) | 0.622 | 1.001(0.998-1.003) | 0.444 |
| **category** |  |  |  |  |
| PCI Failure | 2.334(1.755-3.106) | <0.001 | 2.317(1.740-3.086) | <0.001 |
| TIMI 0 or 1 | 2.716(1.944-3.795) | <0.001 | 2.684(1.919-3.753) | <0.001 |
| RS >50% | 1.705(1.319-2.204) | <0.001 | 1.677(1.297-2.170) | <0.001 |
| Dissection | 1.684(0.899-3.151) | 0.104 | 1.684(0.898-3.157) | 0.104 |
| Thrombosis | 1.732(0.432-6.938) | 0.438 | 1.757(0.438-7.06) | 0.8 |
| Perforation | 1.786(1.121-2.846) | 0.015 | 1.790(1.125-2.863) | 0.014 |
| In hospital MACE | 1.961(0.989-3.883) | 0.054 | 2.012(1.013-3.996) | 0.046 |
|  |  |  |  |  |
| Adjust for Sex Age BMI Dyslipidemia Smoking. MLD, minimal lumen diameter; TIMI, Thrombolysis In Myocardial Infarction; RS, residual stenosis; BMI, Body Mass Index; PCI, percutaneous coronary intervention; OR, odds ratios; CI, confidence intervals. | | | | |

| **Table S7 the relationship between PCI failure and RVD.** | | | | |
| --- | --- | --- | --- | --- |
|  | **Model1** |  | **Model2** |  |
| **continue** | **OR (95%CI)** | **P** | **adjusted OR (95%CI)** | **P** |
| PCI Failure | 0.782(0.625-0.978) | 0.032 | 0.787(0.626-0.987) | 0.039 |
| TIMI 0 or 1 | 1.050(0.932-1.182) | 0.8 | 1.056(0.932-1.198) | 0.389 |
| RS >50% | 0.822(0.647-1.044) | 0.108 | 0.827(0.649-1.054) | 0.126 |
| Dissection | 0.400(0.219-0.732) | 0.003 | 0.391(0.212-0.719) | 0.003 |
| Thrombosis | 1.071(0.736-1.560) | 0.718 | 1.047(0.715-1.533) | 0.813 |
| Perforation | 0.379(0.242-0.595) | <0.001 | 0.382(0.241-0.604) | <0.001 |
| In hospital MACE | 0.285(0.150-0.537) | <0.001 | 0.295(0.152-0.573) | <0.001 |
| **category** |  |  |  |  |
| PCI Failure | 1.917(1.467-2.5-4) | <0.001 | 1.924(1.469-2.519) | <0.001 |
| TIMI 0 or 1 | 1.662(1.188-2.324) | 0.003 | 1.712(1.221-2.402) | 0.002 |
| RS >50% | 2.058(1.535-2.760) | <0.001 | 2.074(1.543-2.789) | <0.001 |
| Dissection | 1.859(0.915-3.777) | 0.086 | 1.878(0.919-3.836) | 0.084 |
| Thrombosis | 0.442(0.116-1.616) | 0.213 | 0.436(0.115-1.651) | 0.222 |
| Perforation | 1.824(1.087-3.063) | 0.023 | 1.778(1.054-2.998) | 0.031 |
| In hospital MACE | 2.505(1.102-5.692) | 0.028 | 2.2630.989-5.178) | 0.053 |
|  |  |  |  |  |
| Adjust for Sex Age BMI Dyslipidemia Smoking. When RVD was analyzed as a category variable, RVD≤ 3.0mm was defined as an event(1). RVD, Reference vessel diameter; MLD, minimal lumen diameter; TIMI, Thrombolysis In Myocardial Infarction; RS, residual stenosis; BMI, Body Mass Index; PCI, percutaneous coronary intervention; OR, odds ratios; CI, confidence intervals. | | | | |
