# Supplementary material for: Prediction of Percutaneous Coronary Intervention Success in Patients With Moderate to Severe Coronary Artery Calcification Using Machine Learning Based on Coronary Angiography: Prospective Cohort Study
Source: J Med Internet Res. 2025 Jul 11;27:e70943. doi: 10.2196/70943 (PMC12274018; doi:10.2196/70943)
Supplement: Multimedia Appendix 2 [file jmir-v27-e70943-s002.pdf]

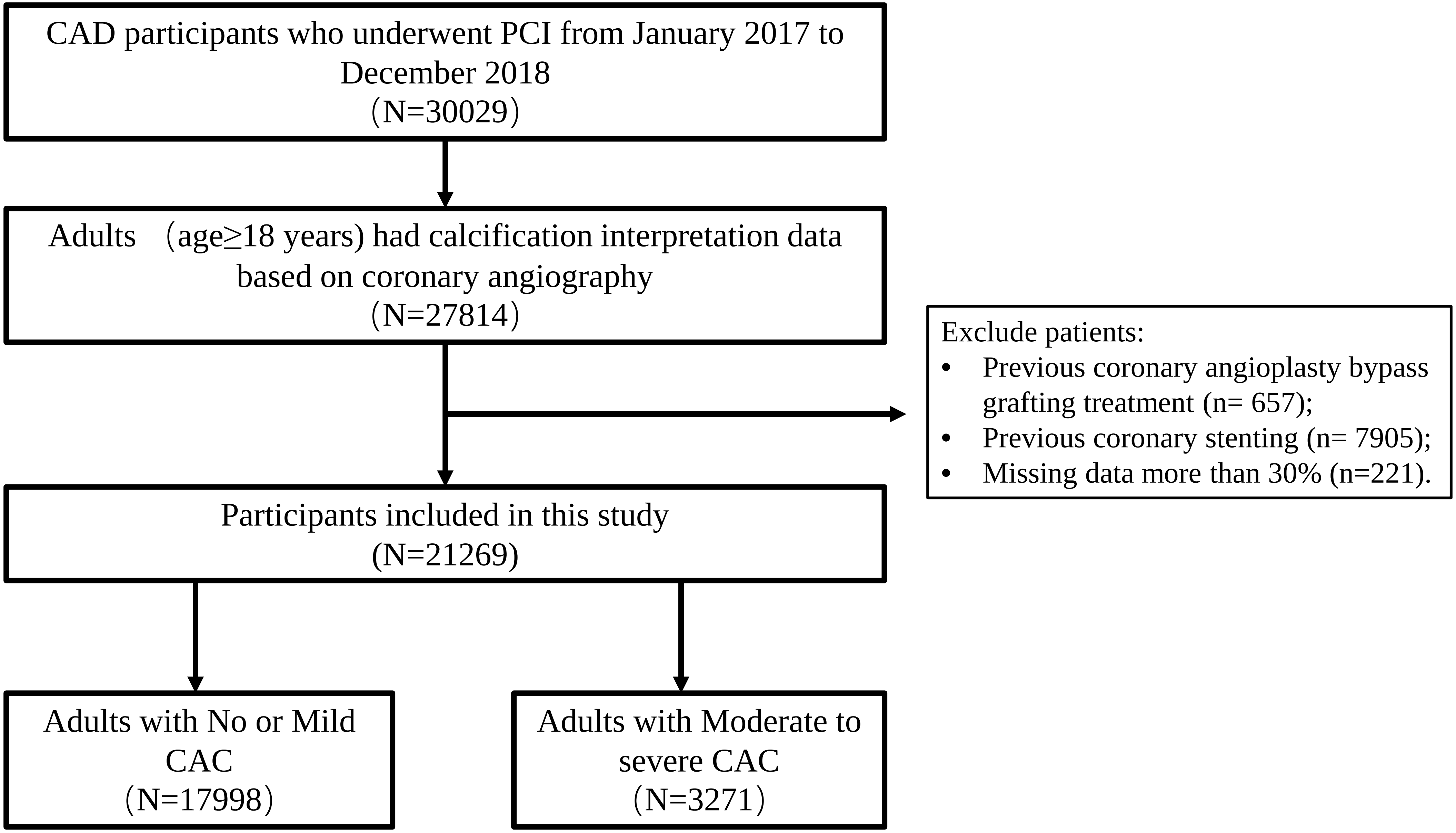

Figure S1 Flowchart of detailed patient selection from 2017-2018 cohort.
PCI, percutaneous coronary intervention; CAD, coronary artery disease; CAC, coronary artery calcification.

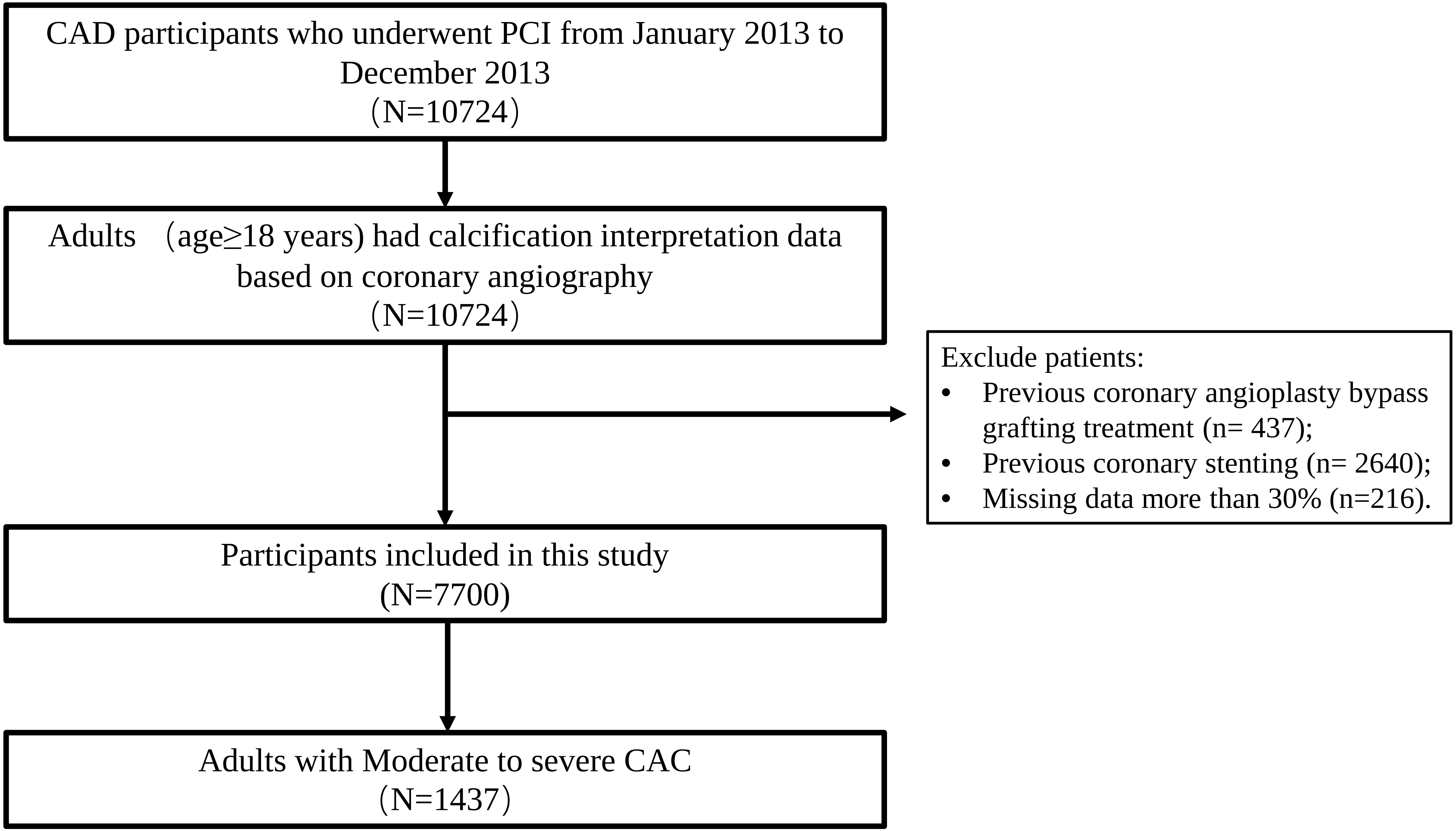

Figure S2 Flowchart of detailed patient selection from 2013 cohort.
PCI, percutaneous coronary intervention; CAD, coronary artery disease; CAC, coronary artery calcification.

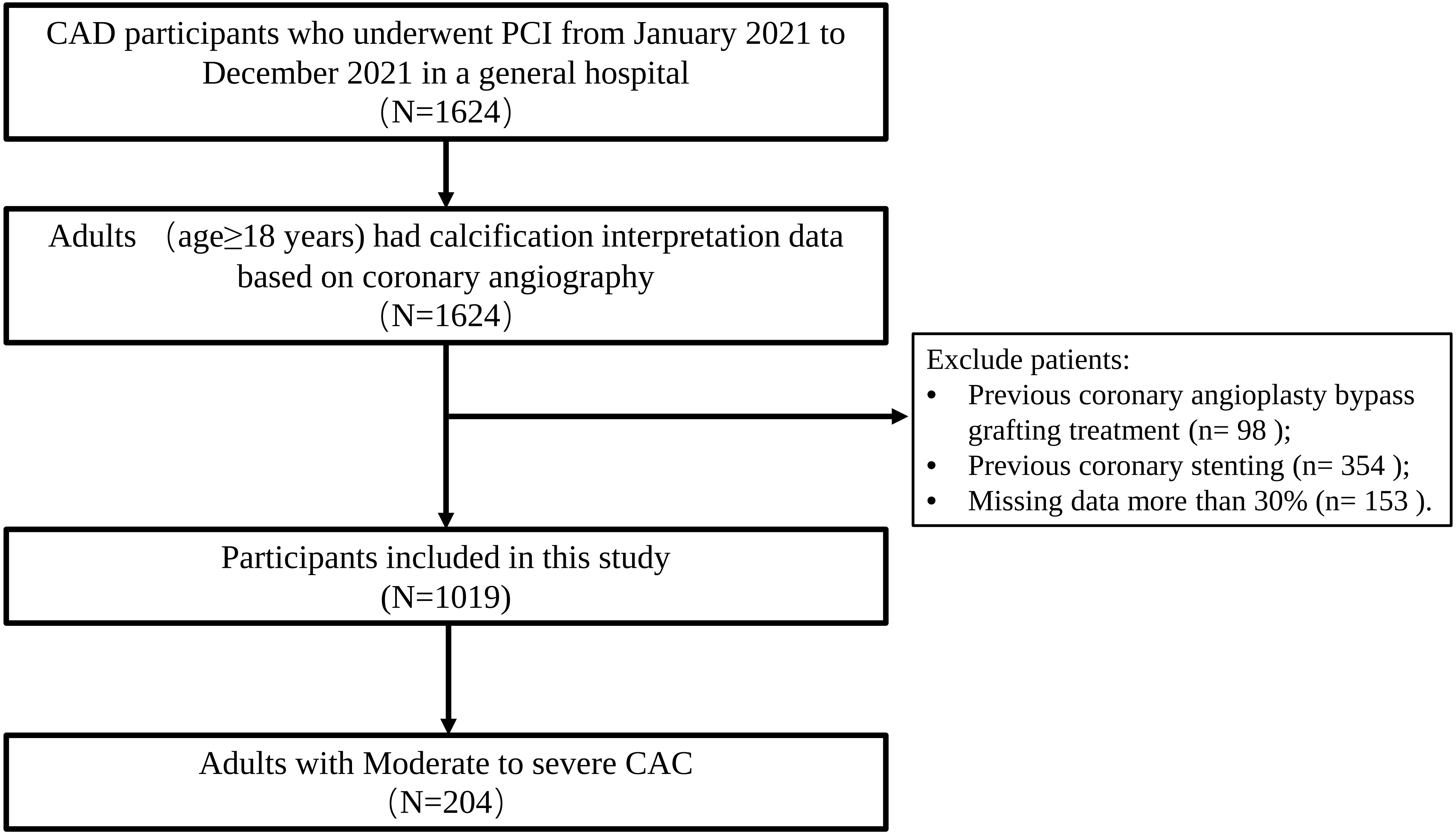

Figure S3 Flowchart of external validation patient selection in a general hospital.
PCI, percutaneous coronary intervention; CAD, coronary artery disease; CAC, coronary artery calcification.

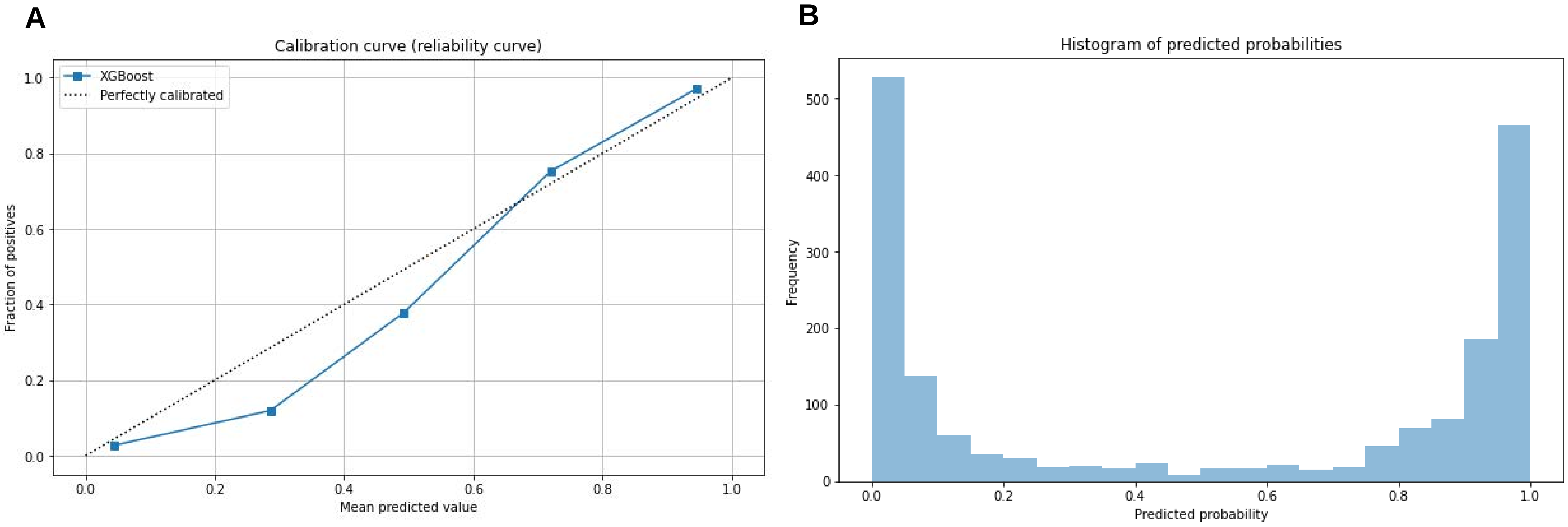

Figure S4 Calibration Curve and Predicted Probability Histogram of the XGBoost Algorithm.

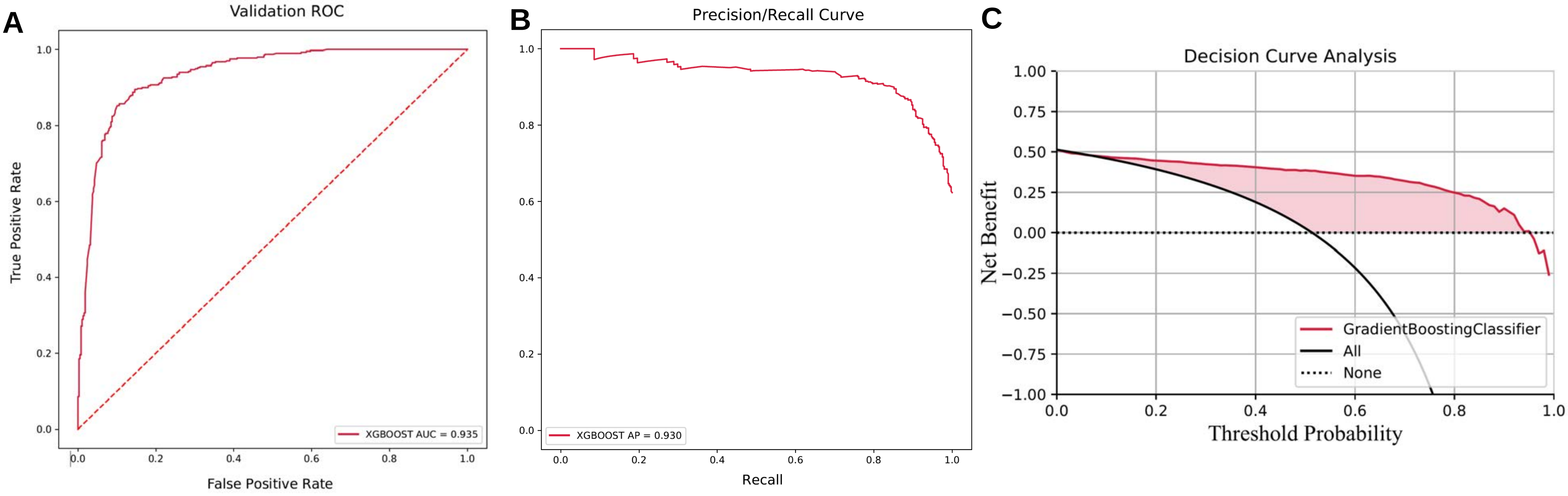

Figure S5 Validation in testing set result. A: ROC of XGBoost; B: P-R curves of XGBoost; C: DCA curve in external validation; DCA, decision curve analysis; ROC, Receiver Operating Characteristic; P-R curve, precision/recall curve.

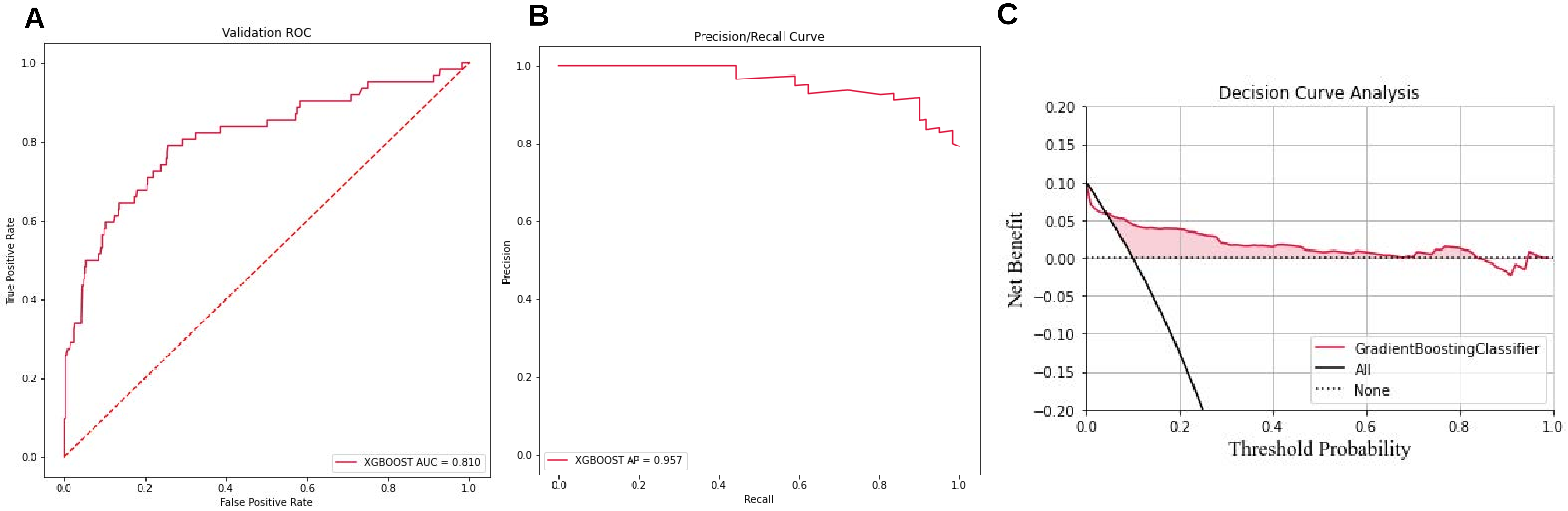

Figure S6 External validation result. A: ROC of XGBoost; B: P-R curves of XGBoost; C: DCA curve in external validation; DCA, decision curve analysis; ROC, Receiver Operating Characteristic; P-R curve, precision/recall curve.

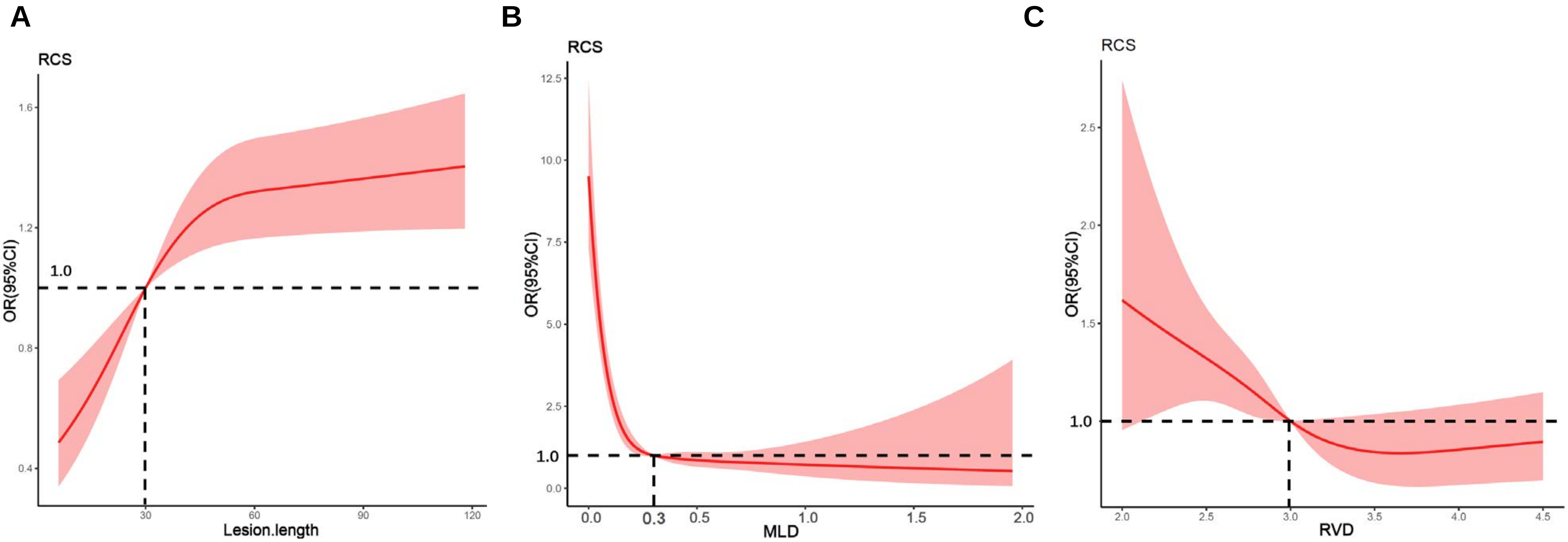

Figure S7 RCS analysis of the relationship between three coronary angiographic characteristics and PCI failure. A: lesion length (cutoff value was 30mmmm); B: MLD (cutoff value was 0.3mm); C: RVD (cutoff value was 3.0mm). RCS, restricted cubic spline; MLD, minimal lumen diameter; PCI, percutaneous coronary intervention; RVD, Reference vessel diameter.

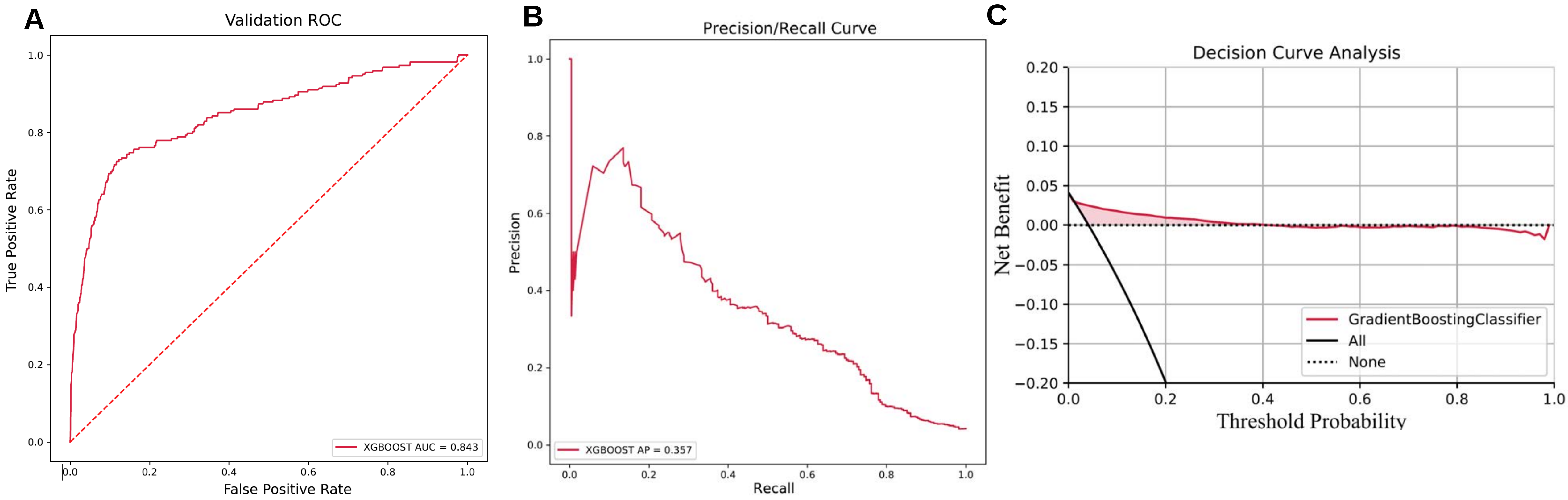

Figure S8 Validation result of simplified XGBoost model in no or mild CAC patients. A: ROC of XGBoost; B: P-R curves of XGBoost; C: DCA curve in validation; DCA, decision curve analysis; ROC, Receiver Operating Characteristic; P-R curve, precision/recall curve.

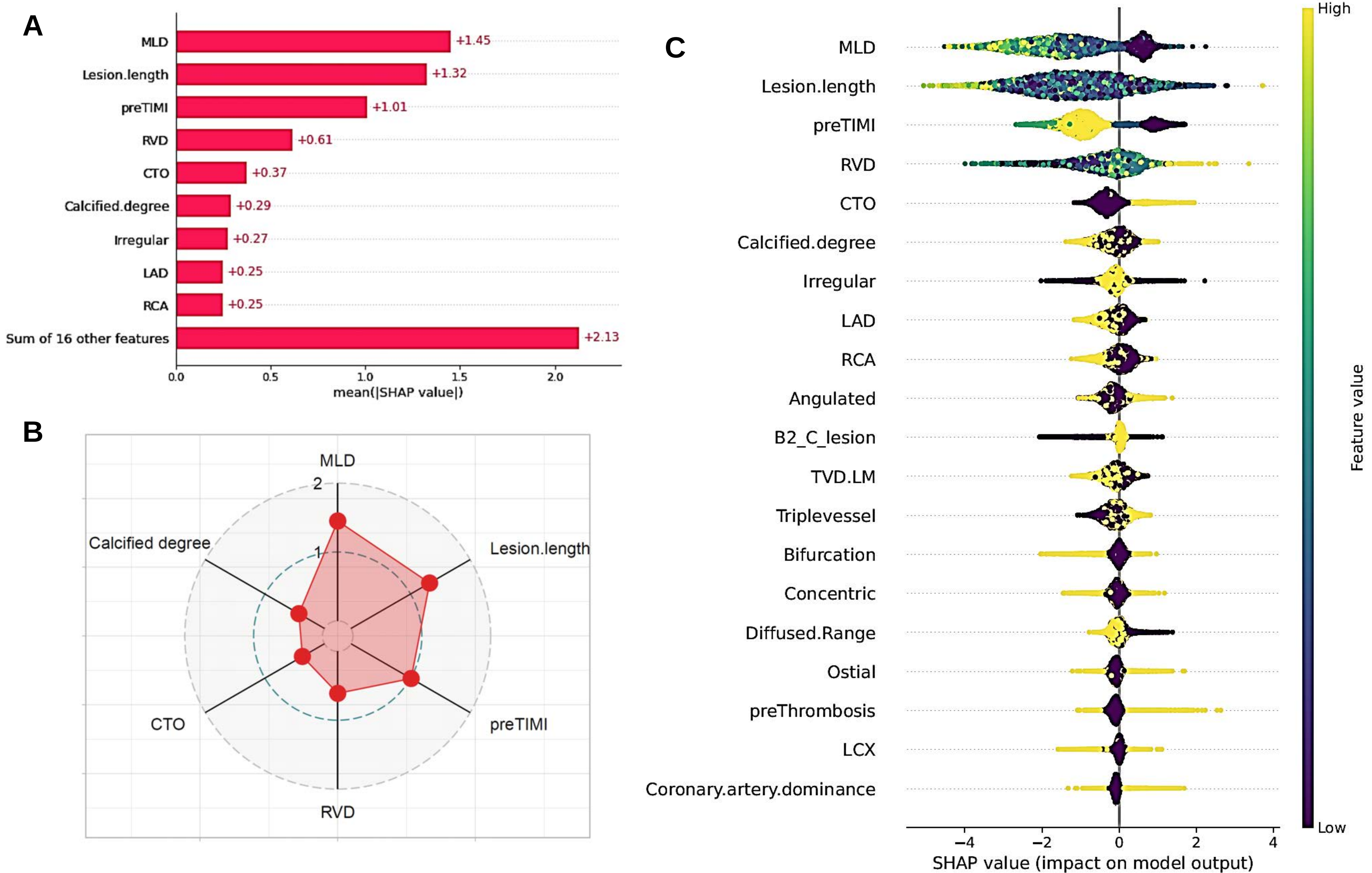
A
MLD
Lesion.length
preTIMI
RVD
CTO
Calcified.degree
Irregular
LAD
RCA
Sum of 16 other features
+1.45
+1.32
+1.01
+0.61
+0.37
+0.29
+0.27
+0.25
+0.25
+2.13
0.0
0.5
1.0
1.5
2.0
mean(|SHAP value|)
B
MLD
2
1
Lesion.length
preTIMI
RVD
CTO
Calcified degree
C
MLD
Lesion.length
preTIMI
RVD
CTO
Calcified.degree
Irregular
LAD
RCA
Angulated
B2_C_lesion
TVD.LM
Triplevessel
Bifurcation
Concentric
Diffused.Range
Ostial
preThrombosis
LCX
Coronary.artery.dominance
−4
−2
0
2
4
SHAP value (impact on model output)
High
Low
Feature value

Figure S9 Visualizing the importance of various predicters by SHAP in no or mild CAC patients. A-B: Bar chart and radar plot that rank the importance of the top 6 significant variables most associated with PCI success rate. C: Impact of top 20 feature in XGBoost model.

RVD, reference vessel diameter; MLD, minimal lumen diameter; CTO, chronic total occlusion; TIMI, Thrombolysis In Myocardial Infarction; LM, left main coronary artery; RCA, right coronary artery; LAD, left anterior descending branch; LCX, left circumflex branch.

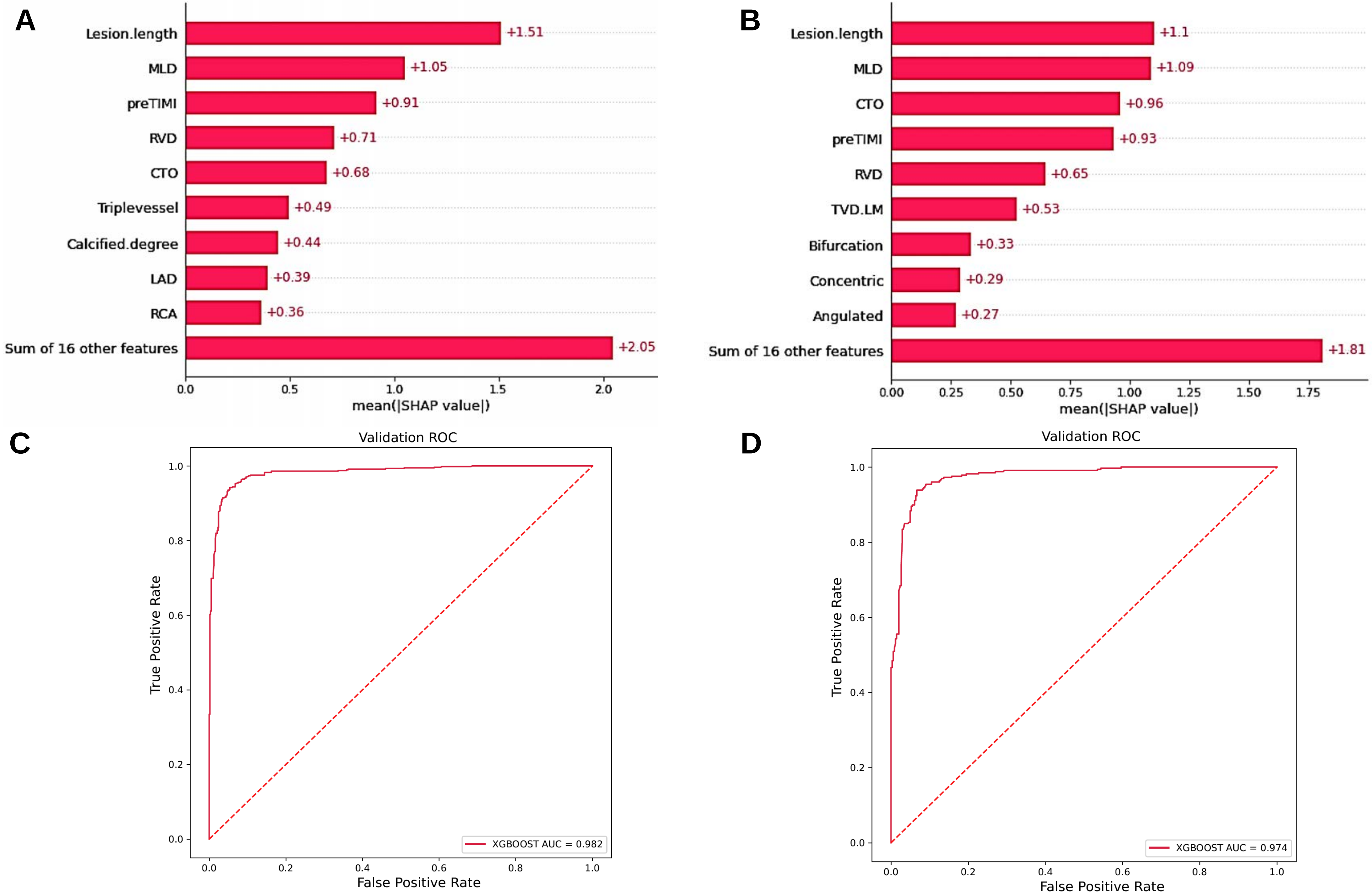
A
Lesion.length +1.51
MLD +1.05
preTIMI +0.91
RVD +0.71
CTO +0.68
Triplevessel +0.49
Calcified.degree +0.44
LAD +0.39
RCA +0.36
Sum of 16 other features +2.05
0.0 0.5 1.0 1.5 2.0
mean(|SHAP value|)
B
Lesion.length +1.1
MLD +1.09
CTO +0.96
preTIMI +0.93
RVD +0.65
TVD.LM +0.53
Bifurcation +0.33
Concentric +0.29
Angulated +0.27
Sum of 16 other features +1.81
0.00 0.25 0.50 0.75 1.00 1.25 1.50 1.75
mean(|SHAP value|)
C
Validation ROC
True Positive Rate
False Positive Rate
XGBOOST AUC = 0.982
D
Validation ROC
True Positive Rate
False Positive Rate
XGBOOST AUC = 0.974

Figure S10 Visualizing the importance of various predicters in ACS patients(A) and non-ACS patients(B). The ROC results of simplified XGBoost model in ACS (C) and non-ACS (D) patients.
ACS, acute coronary syndrome, ROC, Receiver Operating Characteristic; RVD, reference vessel diameter; MLD, minimal lumen diameter; CTO, chronic total occlusion; TIMI, Thrombolysis In Myocardial Infarction; LM, left main coronary artery; RCA, right coronary artery; LAD, left anterior descending branch; LCX, left circumflex branch.
